# Safety outcomes of salbutamol: A systematic review and meta‐analysis

**DOI:** 10.1111/crj.13711

**Published:** 2023-10-16

**Authors:** Lan‐Hong Ma, Li Jia, Ling Bai

**Affiliations:** ^1^ Department of Respiratory, Digestive, and Cardiology Children's Hospital of Xinjiang Uygur Autonomous Region Urumqi China; ^2^ Department of Pharmacy People's Hospital of Xinjiang Uygur Autonomous Region Urumqi China; ^3^ Department of Nephrorheumatology Children's Hospital of Xinjiang Uygur Autonomous Region Urumqi China

**Keywords:** adverse events, meta‐analysis, safety, salbutamol, systematic review

## Abstract

**Purpose:**

Salbutamol has been used to alleviate bronchospasm in airway disease for decades, while its potential risks have not been systematically investigated yet. The risk of any potential adverse events (AEs) in patients treated with salbutamol was assessed through systematic review and meta‐analysis.

**Methods:**

A systematic search of the literature was conducted, using EMBASE, PubMed and Cochrane library, until 3 April 2023. Once the AE incidence was evaluated, randomized controlled trials (RCTs) were eligible for review. The endpoints included the incidence of total AEs, severe AEs, treatment discontinuation and specific AEs. The pooled AEs incidence was analysed via random‐effects model in a single‐arm meta‐analysis. A subgroup study was carried out to examine whether the pooled incidence of AE differed by indications or formulations.

**Results:**

Of the 8912 studies that were identified, 58 RCTs met the inclusion criteria and involved 12 961 participants. The analysis showed the pooled incidences of total AEs, severe AEs and treatment discontinuation in patients treated with salbutamol were 34%, 2% and 3%, respectively. Subgroup analysis indicated that premature labour users and intravenous salbutamol users were more likely associated with total AEs. The most frequently observed specific AEs were palpitations or tachycardia.

**Conclusion:**

This meta‐analysis indicated that salbutamol was associated with a very common risk of palpitations or tachycardia. Clinical vigilance and research efforts are needed to optimize the safe use of salbutamol.

## Graphical abstract

Of the 8912 studies that identified, 58 RCTs met the inclusion criteria and involved 12 961 participants. The pooled incidences of total AEs and severe AEs in patients treated with salbutamol were 34% and 2%, respectively. The most frequently observed specific AEs was palpitations or tachycardia.

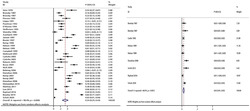

## 1 INTRODUCTION

Salbutamol is the first choice to relieve acute airflow obstruction and symptoms and widely used for asthma, chronic obstructive pulmonary disease (COPD) and other bronchospasm reactions.^1^, ^2^ In the past, numerous studies concluded that the long‐term use of salbutamol was well tolerated.^3^, ^4^, ^5^, ^6^, ^7^ However, Global Initiative for Asthma (GINA) indicated higher use of short‐acting β_2_ agonists (SABA) is associated with higher risk of adverse clinical outcomes, including emergency department presentation^8^ and death.^9^ Although most adverse events (AEs) are manageable and not life‐threatening, they can have a considerable impact on patients' psychological function (such as nervousness and anxiety) or physical function (including palpitation or tachycardia, tremors and headache).^10^, ^11^, ^12^ Furthermore, the safety profiles of salbutamol varied among trials.

To our knowledge, no systematic meta‐analysis focused on the AEs profile of salbutamol has been conducted. To investigate the AE profiles of salbutamol users, we conducted a comprehensive search and meta‐analysis of randomized controlled trials (RCTs).

## 2 METHODS

### 2.1 Data sources, search strategy and selection criteria

The study was carried out and reported in accordance with the Statement of the Preferred Reporting Items for Systematic Reviews and Meta‐Analysis (PRISMA).^13^ RCTs reported the risks of AEs for patients treated with salbutamol were eligible in our study, without any restriction on language. From the beginning to 3 April 2023, a comprehensive search of EmBase, PubMed and the Cochrane library was conducted using the following search terms: ‘salbutamol’, ‘albuterol’, ‘adverse event’, ‘adverse effect’, ‘side effect’, ‘toxicity’, ‘clinical trial’, ‘controlled clinical trial’ and ‘randomized controlled trial’. Only human RCTs were included. When numerous publications of the same clinical trials were identified, only the most comprehensive and latest trial report was included.

Two reviewers worked separately on the literature search and study selection, and any disagreements were resolved through group discussion until a consensus was established. Trials that met the following criteria were included: (a) randomized controlled trials, (b) salbutamol (without restrictions of formulation) was used as the unique medication in the experimental arm, and (c) data on AEs were available. The detail of literature search strategy was shown in Data S1.

### 2.2 Data collection and clinical outcomes

The following data were extracted from each study that met inclusion criteria: the first author's name, publication year, country, number of patients in the salbutamol cohort, mean age, indication, formulation of salbutamol, numbers of patients with total AEs, severe AEs, treatment discontinuation and specific AEs (including tremor, palpitations or tachycardia, headache, nervousness, anxiety, cough, dyspnoea, nausea and vomiting) in the salbutamol cohort and incidence of above AEs in the salbutamol cohort. Two reviewers extracted data separately, and any discrepancies were resolved through consensus.

### 2.3 Quality assessment

Two reviewers independently assessed the methodological quality of the included studies using the Newcastle‐Ottawa Scale (NOS),^14^ by evaluating the following three subscales: (a) selection (four items), (b) comparability (one item) and (c) outcome (three items). Items that are judged as adequate receive a star and 1 point. Responses are summed (range 0 to 9). Studies scoring 7 or over are considered of good quality, 5 or 6 of fair quality and 0 to 4 of poor quality. Any disagreements were settled through consensus.

### 2.4 Statistical analysis

The pooled incidence of total AEs, severe AEs, treatment discontinuation and specific AEs (including tremor, palpitations or tachycardia, headache, nervousness, anxiety, cough, dyspnoea, nausea and vomiting) after salbutamol treatment were the endpoint of this meta‐analysis. The number of total AEs, severe AEs, treatment discontinuations and specific AEs from the salbutamol treated group in each study were extracted to calculate the incidence. A random‐effects model was used to generate the pooled incidence and the corresponding 95% confidence interval (CI).^15^, ^16^ The *I* ^2^ and *Q* statistic were used to measure heterogeneity. The *I* ^2^ > 50.0% or the *P*_*Q statistic*_ < 0.10 was used to characterize significant heterogeneity.^17^, ^18^ The potential impact of a single trial on pooled incidences of total AEs, severe AEs and treatment discontinuation was assessed by sensitivity analysis.^19^ The researchers used subgroup analysis to evaluate whether the pooled incidences of total AEs, severe AEs and treatment discontinuation varied by indications (premature labour, asthma or COPD) and the formulations of salbutamol (inhaled, oral or intravenous). The interaction *P* test was used to assess the subgroup analysis.^20^ A two‐sided *P* value lower than 0.05 was considered statistically significant. Publication bias was conducted using funnel plots, Egger's test and Begg's test.^21^, ^22^, ^23^ All analyses were performed by STATA 10.0 (Stata Corporation, College Station, TX, USA).

## 3 RESULTS

### 3.1 Literature search

**FIGURE 1:**
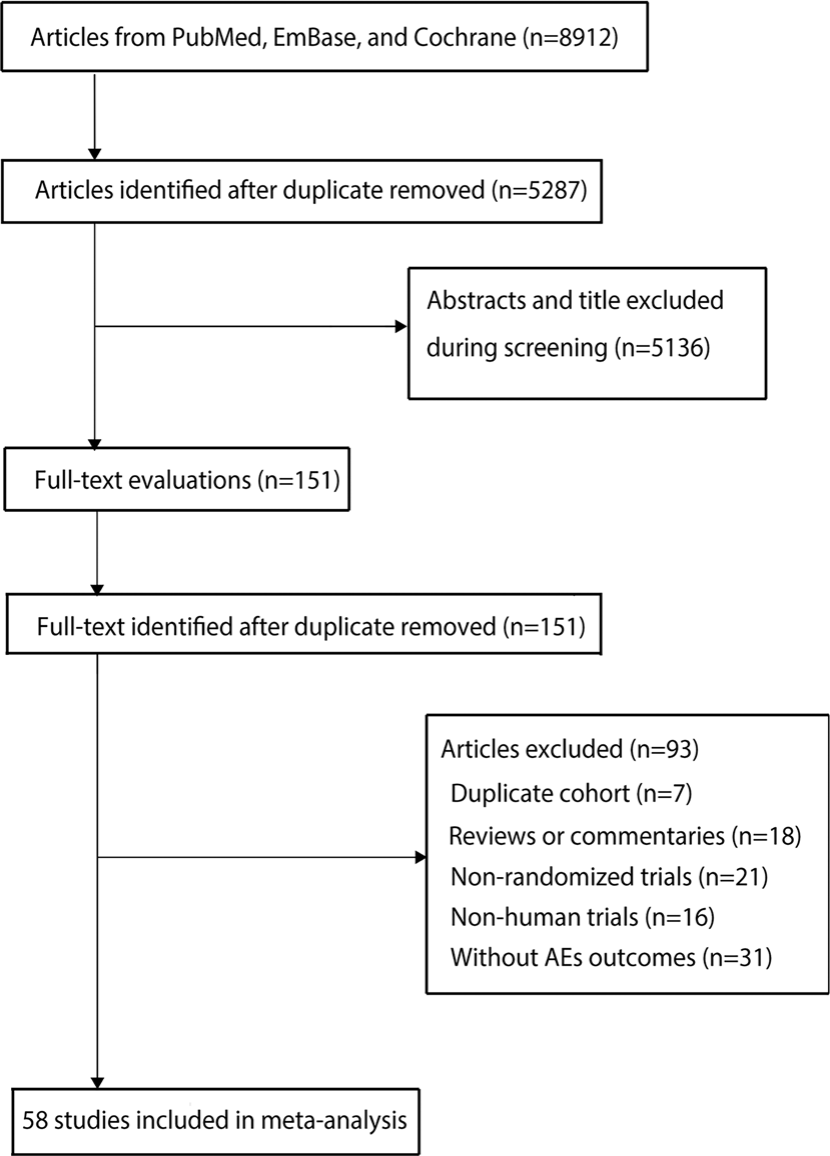
The flow chart of study selection.

The initial search yielded 8912 potentially relevant trails. After duplicate publications were removed, 5287 trails were included. After checking the titles and abstracts, 5136 records were eliminated, leaving 151 possibly relevant articles to be reviewed in full text. Based on the full‐text review, 93 articles were ruled out for one or more of the following reasons: reviews, duplicated cohorts, commentaries, non‐randomized trials, non‐human trials or without AEs outcomes. Ultimately, 58 RCTs with a total of 12 961 patients were included in this meta‐analysis.^24^, ^25^, ^26^, ^27^, ^28^, ^29^, ^30^, ^31^, ^32^, ^33^, ^34^, ^35^, ^36^, ^37^, ^38^, ^39^, ^40^, ^41^, ^42^, ^43^, ^44^, ^45^, ^46^, ^47^, ^48^, ^49^, ^50^, ^51^, ^52^, ^53^, ^54^, ^55^, ^56^, ^57^, ^58^, ^59^, ^60^, ^61^, ^62^, ^63^, ^64^, ^65^, ^66^, ^67^, ^68^, ^69^, ^70^, ^71^, ^72^, ^73^, ^74^, ^75^, ^76^, ^77^, ^78^, ^79^, ^80^, ^81^ The selection procedure is depicted in Figure 1.

### 3.2 Study characteristics

Summary of the main characteristics of the included studies was displayed in Table S1. Of the included 58 trials, the number of salbutamol users ranged from 11 to 8393, which were published from 1978 to 2020,^24^, ^25^, ^26^, ^27^, ^28^, ^29^, ^30^, ^31^, ^32^, ^33^, ^34^, ^35^, ^36^, ^37^, ^38^, ^39^, ^40^, ^41^, ^42^, ^43^, ^44^, ^45^, ^46^, ^47^, ^48^, ^49^, ^50^, ^51^, ^52^, ^53^, ^54^, ^55^, ^56^, ^57^, ^58^, ^59^, ^60^, ^61^, ^62^, ^63^, ^64^, ^65^, ^66^, ^67^, ^68^, ^69^, ^70^, ^71^, ^72^, ^73^, ^74^, ^75^, ^76^, ^77^, ^78^, ^79^, ^80^, ^81^ and 38 (65.5%), seven (12.1%), five (8.6%) and eight (13.8%) focused on asthma, COPD, premature labour and other bronchoconstriction indications, respectively. Inhaled salbutamol was used in 75.9% of trails (44/58), while oral salbutamol was used in seven trails, and intravenous salbutamol was used in the rest seven trails. Salbutamol's dosage ranged from 200 μg to 30 mg per day, with treatment duration ranging from 1 day to 52 weeks.

### 3.3 Quality of the studies and publication bias

Table S2 indicates the methodological quality of the included studies. Thirty‐one studies reported six stars and the remaining 27 studies with five stars. Furthermore, visual inspection of the funnel plots and Egger's test revealed no indication of publication bias for severe AEs (Figures S1–S12). Similar to the Egger's test, the Begg's test also presented no evidence of asymmetry.

### 3.4 Incidences of Total AEs

**FIGURE 2:**
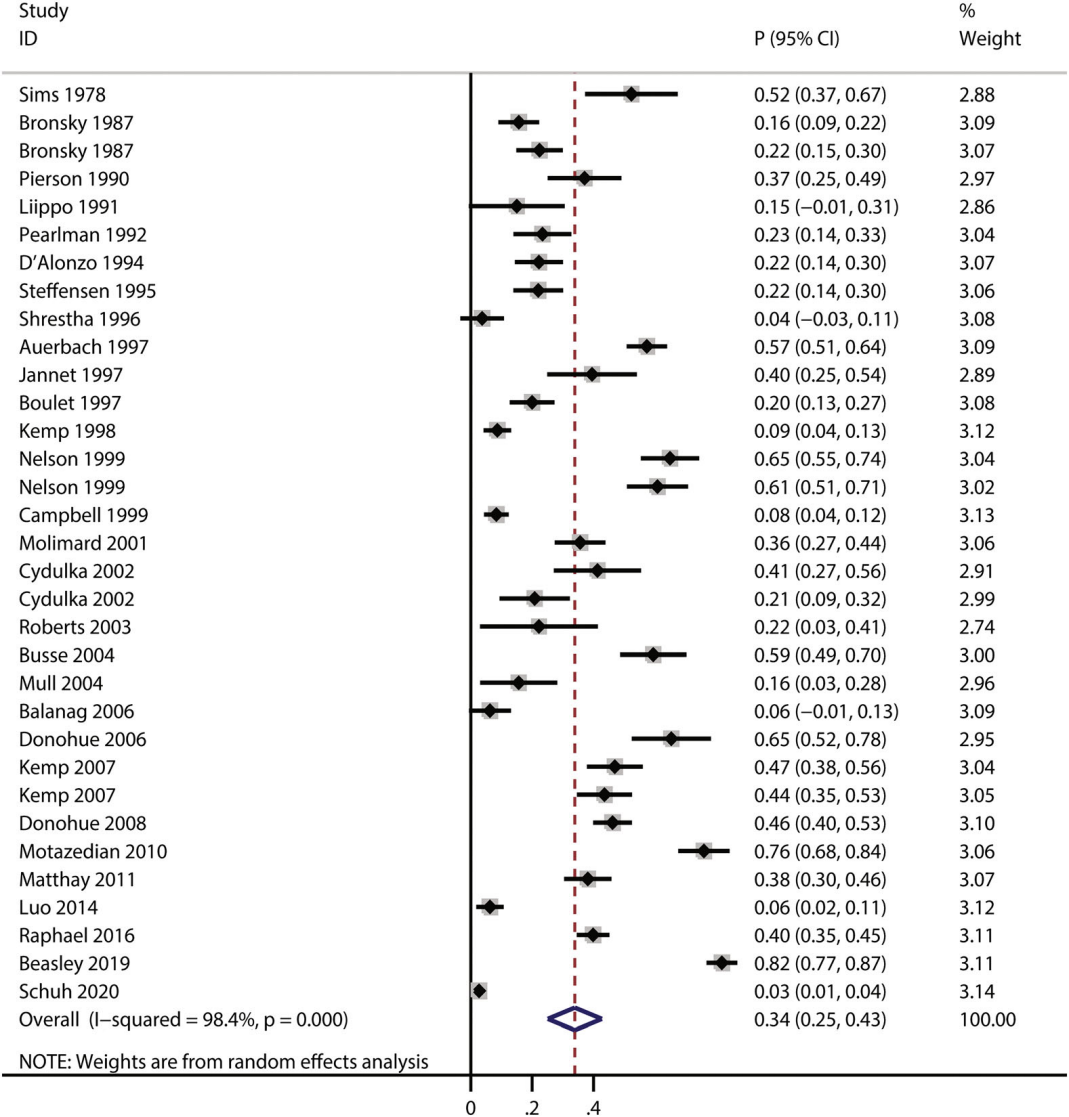
Forest plot for the pooled incidence of total AEs.

A total of 30 trails reported the risk of total AEs for patients treated with salbutamol,^24^, ^26^, ^29^, ^31^, ^32^, ^34^, ^35^, ^38^, ^40^, ^41^, ^42^, ^45^, ^49^, ^50^, ^54^, ^55^, ^56^, ^57^, ^58^, ^59^, ^63^, ^64^, ^65^, ^66^, ^69^, ^72^, ^76^, ^77^, ^79^, ^81^ and the pooled incidence of total AEs was 34% (*95% CI*: 25%–43%, *I* ^2^ = 98.4%, *P* < 0.001; Figure 2). The incidences of total AEs varied in different indication subgroups and formulation subgroups (Table 1). As for indication subgroups, there were three studies,^24^, ^42^, ^69^ 19 studies^26^, ^29^, ^31^, ^32^, ^34^, ^38^, ^41^, ^45^, ^50^, ^54^, ^56^, ^57^, ^58^, ^63^, ^65^, ^76^, ^77^, ^79^, ^81^ and five studies^35^, ^40^, ^49^, ^64^, ^66^ reported total AEs in premature labour subgroup, asthma subgroup and COPD subgroup, and the pooled incidence was highest in premature labour subgroup (pooled incidence: 57%, *95% CI*: 0.33–0.80, *I* ^*2*^ *=* 90.4%, *P <* 0.001). As for formulation subgroups, total AEs were reported in 24 studies,^26^, ^31^, ^32^, ^34^, ^35^, ^38^, ^40^, ^41^, ^45^, ^49^, ^50^, ^54^, ^56^, ^58^, ^59^, ^63^, ^64^, ^65^, ^66^, ^72^, ^76^, ^77^, ^79^, ^81^ four studies^24^, ^42^, ^57^, ^69^ and two studies^29^, ^55^ in inhaled, intravenous and oral subgroup, while the pooled incidence was highest in intravenous subgroup (pooled incidence: 51%, *95% CI*: 0.21–0.81, *I* ^2^ *=* 92.9%, *P <* 0.001).

**TABLE 1:** Subgroup analyses for adverse events.

Outcomes	Subgroup		No. of studies	Incidence (95% CI)	*I* ^2^ (%)	*P*_Q statistic_	*P* value between subgroups
Total AEs	Different indications	Premature labour	3	0.57 (0.33–0.80)	90.4	<0.001	<0.001
		Asthma	19	0.30 (0.20–0.40)	98.4	<0.001	
		COPD	5	0.38 (0.16–0.60)	98.1	<0.001	
	Different formulations	Inhaled	24	0.33 (0.24–0.43)	98.5	<0.001	<0.001
		Oral	2	0.22 (0.08–0.36)	90.6	<0.001	
		Intravenous	4	0.51 (0.21–0.81)	92.9	<0.001	
Severe AEs	Different indications	Premature labour	0	‐	‐	‐	0.004
		Asthma	5	0.02 (0.01–0.03)	59.2	0.023	
		COPD	1	0.06 (0.03–0.09)	‐	‐	
	Different formulations	Inhaled	6	0.02 (0.01–0.03)	67.6	0.003	0.004
		Oral	0	‐	‐	‐	
		Intravenous	1	0.06 (0.02–0.09)	‐	‐	
Treatment discontinuations	Different indications	Premature labour	0	‐	‐	‐	0.001
		Asthma	7	0.01 (0.01–0.02)	0	0.436	
		COPD	3	0.13 (0.02–0.23)	88.7	<0.001	
	Different formulations	Inhaled	11	0.03 (0.01–0.04)	70.1	<0.001	0.108
		Oral	1	0.05 (−0.01–0.12)	‐	‐	
		Intravenous	0	‐	‐	‐	

Abbreviations: AEs, adverse events; CI, confidence interval; COPD, chronic obstructive pulmonary disease.

### 3.5 Incidences of severe AEs

**FIGURE 3:**
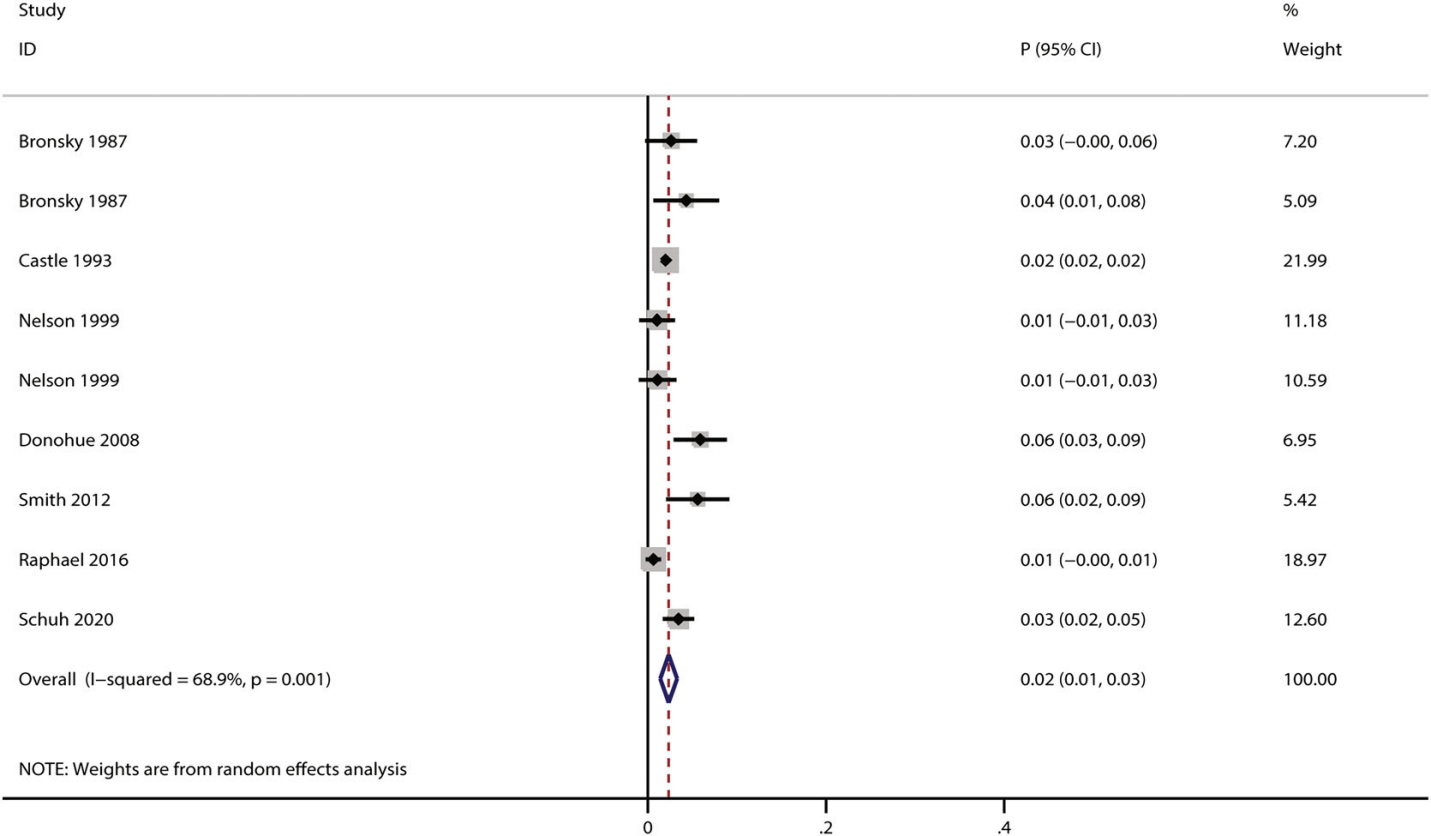
Forest plot for the incidence of severe AEs.

Seven trials reported the risk of severe AEs in salbutamol users,^26^, ^33^, ^50^, ^66^, ^73^, ^77^, ^81^ and the pooled incidence was 2% (*95% CI*: 1%–3%, *I* ^2^ = 68.9%, *P* < 0.001; Figure 3). The incidences of severe AEs varied in different indication subgroups and formulation subgroups (Table 1). Compared with COPD subgroup (pooled incidence: 6%, *95% CI*: 3%–9%),^66^ the risk of severe AEs in asthma subgroup was slightly lower (pooled incidence: 2%, *95% CI*: 1%–3%, *I* ^*2*^ = 59.2%, *P* = 0.004),^26^, ^33^, ^50^, ^77^, ^81^ while inhaled salbutamol subgroup (pooled incidence: 2%, *95% CI*: 1%–3%, *I* ^*2*^ = 67.6%, *P* = 0.004)^26^, ^33^, ^50^, ^66^, ^77^, ^81^ was with a lower risk of severe AEs than intravenous subgroup (pooled incidence: 6%, *95% CI*: 2%–9%, *P* = 0.038).^73^ No studies reported severe AEs either in premature labour subgroup or oral subgroup.

### 3.6 Incidences of treatment discontinuations

**FIGURE 4:**
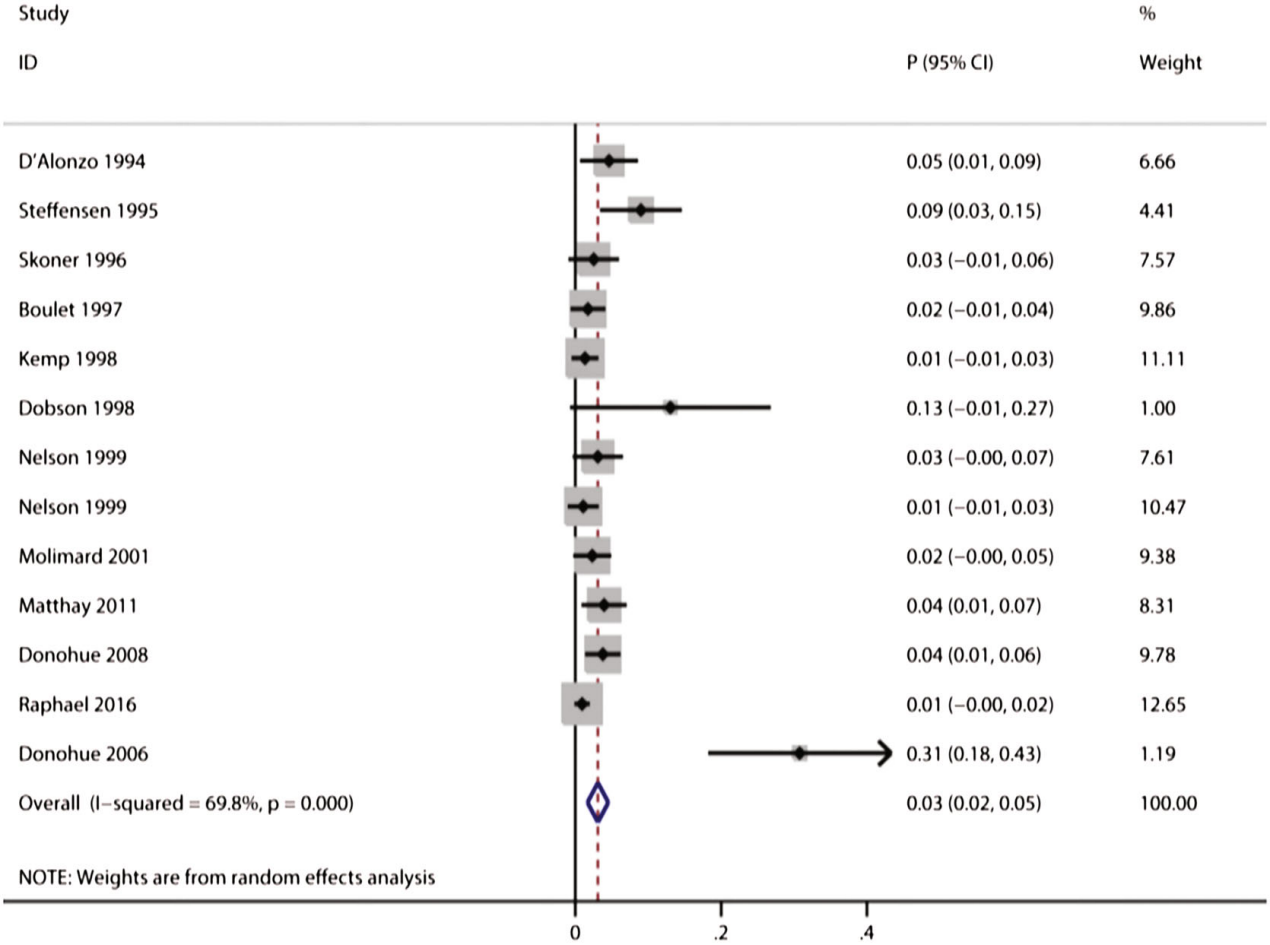
Forest plot for the incidence of treatment discontinuation.

Twelve trials reported the risk of treatment discontinuations in salbutamol users,^34^, ^35^, ^39^, ^41^, ^44^, ^45^, ^50^, ^54^, ^64^, ^66^, ^72^, ^77^ and the pooled incidence of treatment discontinuations was 3% (*95%CI*: 2%–5%, *I* ^2^ = 69.8%, *P* < 0.001; Figure 4). There was a higher risk of treatment discontinuation in COPD subgroup (pooled incidence: 13%, *95% CI*: 2%–23%, *I* ^2^ = 88.7%)^35^, ^64^, ^66^ than asthma subgroup (pooled incidence: 1%, *95% CI*: 1%–2%, *I* ^*2*^ = 0%, *P* < 0.001).^34^, ^39^, ^41^, ^45^, ^50^, ^54^, ^77^ There are no significant differences between oral subgroup and inhaled subgroup (*P* = 0.108, Table 1). No studies reported treatment discontinuations in either premature labour subgroup or intravenous subgroup.

### 3.7 Incidences of specific AEs

#### 3.7.1 Cardiovascular system

The pooled incidence of palpitations or tachycardia was 16% (*95%CI*: 11%–22%, *I* ^2^ = 98.7%, *P* < 0.001; Figure S13).

#### 3.7.2 Nervous system

The pooled incidences of tremor, headache, nervousness and anxiety were 9% (*95%CI*: 7%–11%, *I* ^*2*^ = 87%, *P* < 0.001), 8% (*95%CI*: 6%–10%, *I* ^2^ = 88.2%, *P* < 0.001), 5% (*95%CI*: 3%–7%, *I* ^*2*^ = 44.1%, *P* = 0.057) and 13% (*95%CI*: 6%–21%, *I* ^2^ = 92.3%, *P* < 0.001), respectively (Figures S14–S17).

#### 3.7.3 Respiratory system

The pooled incidences of cough and dyspnoea were 3% (*95%CI*: 2%–5%, *I* ^*2*^ = 51.5%, *P* = 0.036) and 7% (*95%CI*: 2%–12%, *I* ^2^ = 86.7%, *P* < 0.001), respectively (Figures S18 and S19).

#### 3.7.4 Digestive system

The pooled incidences of nausea and vomiting were 5% (*95%CI*: 3%–8%, *I* ^2^ = 81.7%, *P* < 0.001) and 7% (*95%CI*: 3%–11%, *I* ^2^ = 78.8%, *P* < 0.001), respectively (Figures S20 and S21).

### 3.8 Sensitivity analysis

A sensitivity analysis was conducted if any AE outcome with significant heterogeneity in the reported results. All the results (total AEs, severe AEs, treatment discontinuation and all specific AEs) were not significantly altered by sequentially omitting each study in the meta‐analysis (Figures S22–S24).

## 4 DISCUSSION

Nowadays, numerous studies have already illustrated the treatment effectiveness of salbutamol for patients undergoing various conditions,^82^, ^83^, ^84^ while no study systematically assessed the pooled effect estimates for AEs. This meta‐analysis involved 12 961 patients treated with salbutamol from 58 RCTs across different indications and different salbutamol formulations. The risk of total AEs was very common (≥1/10) as 34 per 100 patients in salbutamol users, while the risk of severe AEs and treatment discontinuation were common (2% and 3%, respectively). The most frequent specific AEs were palpitations or tachycardia (16%).

As for different indications, premature labour users (57%) were more likely to be associated with total AEs. Compared with inhaled salbutamol users, intravenous users were more likely to be associated with total AEs (51% vs. 33%, *P* < 0.001) and severe AEs (6% vs. 2%, *P* = 0.004), while the rates of treatment discontinuation were 3% and 5% in inhaled users and oral salbutamol users, with no significant differences (*P* = 0.108). Sriprasart et al.^85^ conducted a meta‐analysis in asthmatic patients (aged ≥12 years old) in inhaled SABA users, which reported the rates of treatment discontinuation, and severe AEs were 3.9% and 1.8%, respectively. Our results were consistent with previous systematic reviews. Therefore, additional effective treatment strategies should be applied to these salbutamol users to prevent further AEs risk.

Palpitations or tachycardia is the highest incidence in our study. It is also a common adverse reaction in clinical practice, which could be explained by the following reason: (1) Hypokalaemia or prolonged QT interval could cause arrhythmogenic effects; (2) the increased heart rate could cause by shortened diastole; (3) increased sympathetic outflow are significantly associated with poor prognosis of cardiovascular disease and COPD.^86^, ^87^, ^88^, ^89^ In a retrospective study involving 64 patients with asthma treated with salbutamol, 95% of the patients developed tachycardia.^90^ The results of another retrospective database analysis showed that the incidence of supraventricular tachycardia caused by salbutamol treatment in children with asthma from 0 to 18 years old from 2006 to 2015 was 0.39‰.^91^ Previous studies and our meta‐analysis indicated the use of salbutamol in patients with cardiovascular disease should be more carefully.

As for other specific AEs, salbutamol seems to have a greater effect on the nervous system, while the pooled incidence of anxiety and tremor was 13% and 9%, respectively. It is worth noting that not all the labels for salbutamol suggest the adverse effects on anxiety. A prospective study reported that anxiety has been associated with increased asthma‐related exacerbations.^92^ However, whether the anxiety caused by the use of salbutamol affects the disease state needs further analysis. In addition, salbutamol could induce skeletal muscle tremor, irrespective of short‐ or long‐term use of salbutamol.^93^, ^94^ Tremor is usually transient, that is, disappears after stopping the use of salbutamol, and usually does not require additional treatment. Considering that patients often feel worried or fearful, it is suggested that clinicians can provide appropriate emotional comfort when patients experience such adverse events.

These results are important because the overprescription of SABA continues to increase worldwide especially in asthmatic patients, which is associated with an increased risk of asthma exacerbations, mortality and incremental health care resource utilization.^95^, ^96^ However, it is important to note that this meta‐analysis has limitations, mainly due to the variation in study design features (such as duration of follow‐up), features of the population (such as severity of illness, age and gender) and intervention factors (such as dosage, timing or duration of treatment). These may be the determinant reasons for the high heterogeneity of results we observed in all of the quantitative analyses, which could not be interpreted by sensitivity and subgroup analyses. And we performed the risk of bias assessment and revealed all of the included studies with fair quality. What is more, our study mainly focuses on mono‐therapy with salbutamol. Furthermore, some of the included RCTs had small sample sizes and varied study duration. More studies with sufficient methodological quality are needed to monitor the safety of salbutamol. Because of the mentioned considerations, our pooled estimate of incidence might be underestimated.

## 5 CONCLUSION

In conclusion, the risk of total AEs may be quite common in the clinical use of salbutamol. Incidences of severe AEs and treatment discontinuation are also considered common. When using salbutamol in clinical practice, more attention is needed to be paid to palpitation or tachycardia, which might occur more frequently. Moreover, premature labour users or intravenous salbutamol users might have a higher risk of AEs, which should be cautious in clinical practice. For the safe use of salbutamol, further research is needed, particularly large‐scale intensive monitoring studies.

## AUTHOR CONTRIBUTIONS

We declare that this work was done by the authors named in this article, and all liabilities pertaining to claims relating to the content of this article will be borne by the authors. Ling Bai conceived and designed the study; Lanhong Ma, Li Jia and Ling Bai performed the study; Lanhong Ma analysed the data; Ling Bai contributed to reagents/materials/analysis tools; Lanhong Ma wrote the manuscript.

## CONFLICT OF INTEREST STATEMENT

No conflict of interest was associated with this work.

## ETHICS STATEMENT

No ethical approval was required for this systematic review and meta‐analysis.

## Supporting information


**Figure S1.** Funnel plot for the incidence of total AEs.

**Figure S2.** Funnel plot for the incidence of severe AEs.

**Figure S3.** Funnel plot for the incidence of treatment discontinuation.

**Figure S4.** Funnel plot for the incidence of tremor.

**Figure S5.** Funnel plot for the incidence of palpitations or tachycardia.

**Figure S6.** Funnel plot for the incidence of headache.

**Figure S7.** Funnel plot for the incidence of nervousness.

**Figure S8.** Funnel plot for the incidence of anxiety.

**Figure S9.** Funnel plot for the incidence of cough.

**Figure S10.** Funnel plot for the incidence of dyspnoea.

**Figure S11.** Funnel plot for the incidence of nausea.

**Figure S12.** Funnel plot for the incidence of vomiting.

**Figure S13.** Forest plot for the incidence of palpitations or tachycardia.

**Figure S14.** Forest plot for the incidence of tremor.

**Figure S15.** Forest plot for the incidence of headache.

**Figure S16.** Forest plot for the incidence of nervousness.

**Figure S17.** Forest plot for the incidence of anxiety.

**Figure S18.** Forest plot for the incidence of cough.

**Figure S19.** Forest plot for the incidence of dyspnoea.

**Figure S20.** Forest plot for the incidence of nausea.

**Figure S21.** Forest plot for the incidence of vomiting.

**Figure S22.** Sensitivity analysis for the incidence of total AEs.

**Figure S23.** Sensitivity analysis for the incidence of severe AEs.

**Figure S24.** Sensitivity analysis for the incidence of treatment discontinuation.

**Data S1.** Supporting Information.

**Table S1.** The baseline characteristics of identified trials and patients.

**Table S2.** Risk of bias of the included trials.

## Data Availability

The data that support the findings of this study are available from the corresponding author upon reasonable request.
