# Supplementary figures and images for: Safety outcomes of salbutamol: A systematic review and meta‐analysis
Source: Clin Respir J. 2023 Oct 16;17(12):1254–64. doi: 10.1111/crj.13711 (PMC10730473; doi:10.1111/crj.13711)

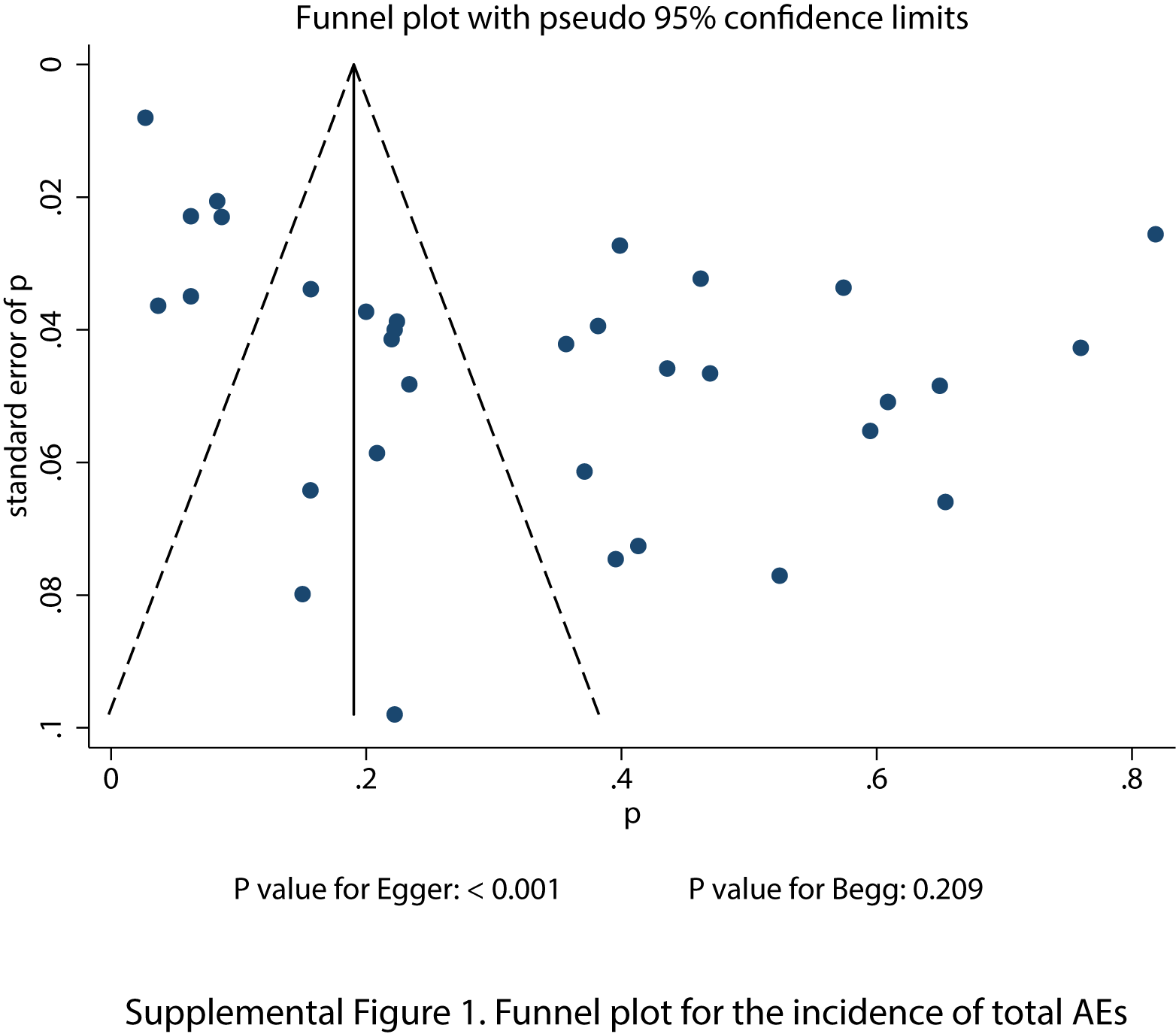

Supplement: Supplementary file 1 — Figure S1. Funnel plot for the incidence of total AEs. [file CRJ-17-1254-s026.tif]

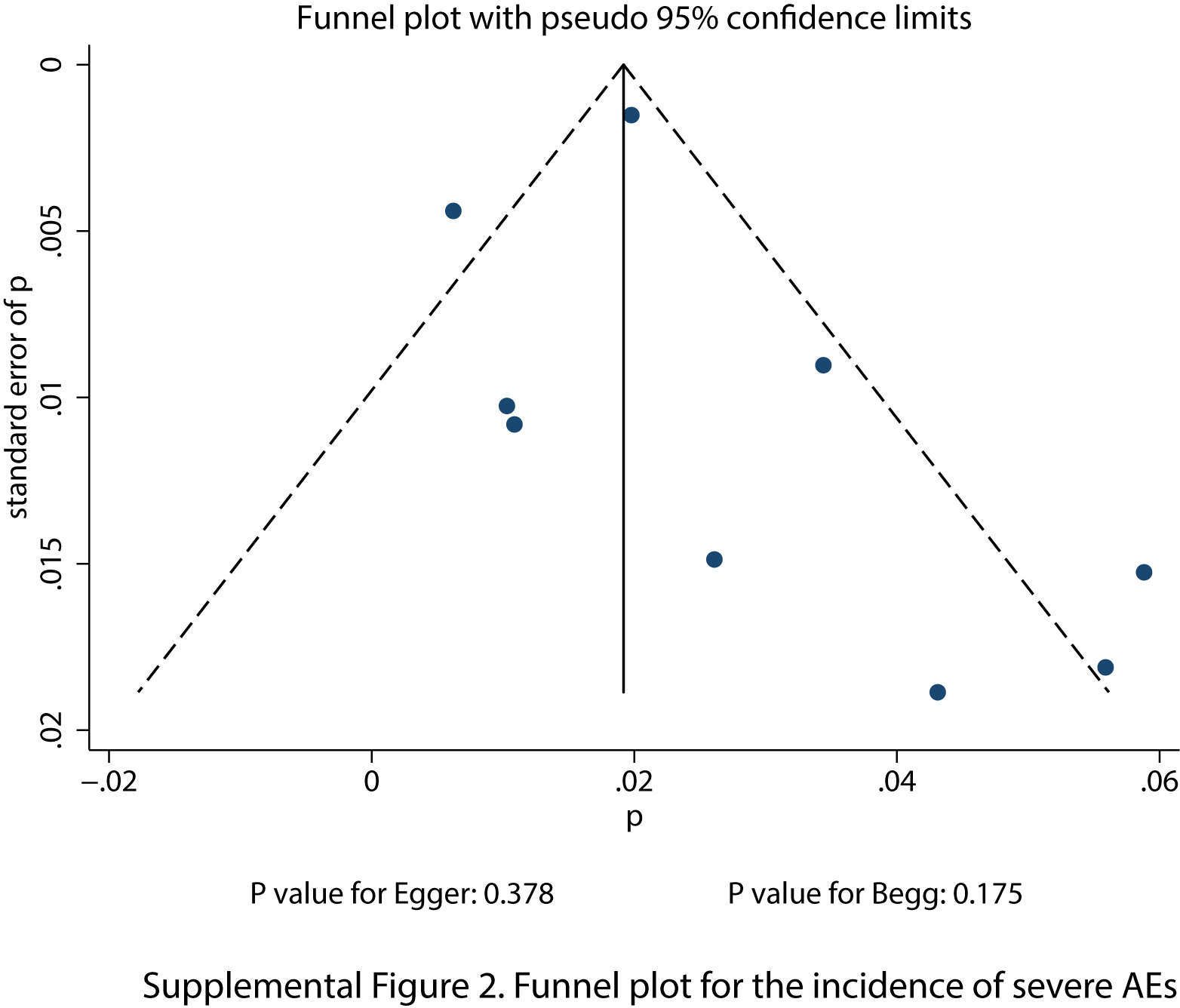

Supplement: Supplementary file 2 — Figure S2. Funnel plot for the incidence of severe AEs. [file CRJ-17-1254-s002.tif]

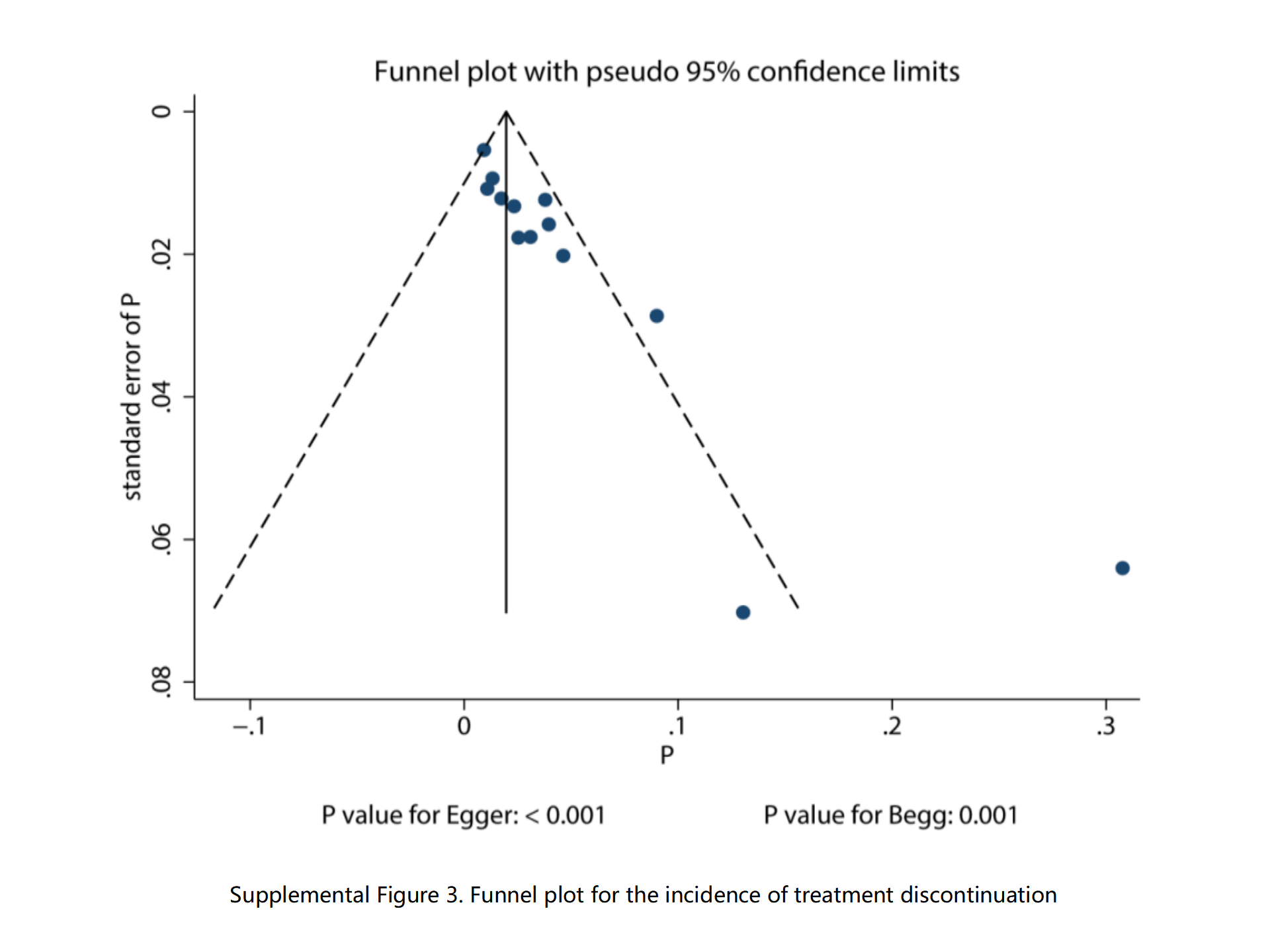

Supplement: Supplementary file 3 — Figure S3. Funnel plot for the incidence of treatment discontinuation. [file CRJ-17-1254-s009.tif]

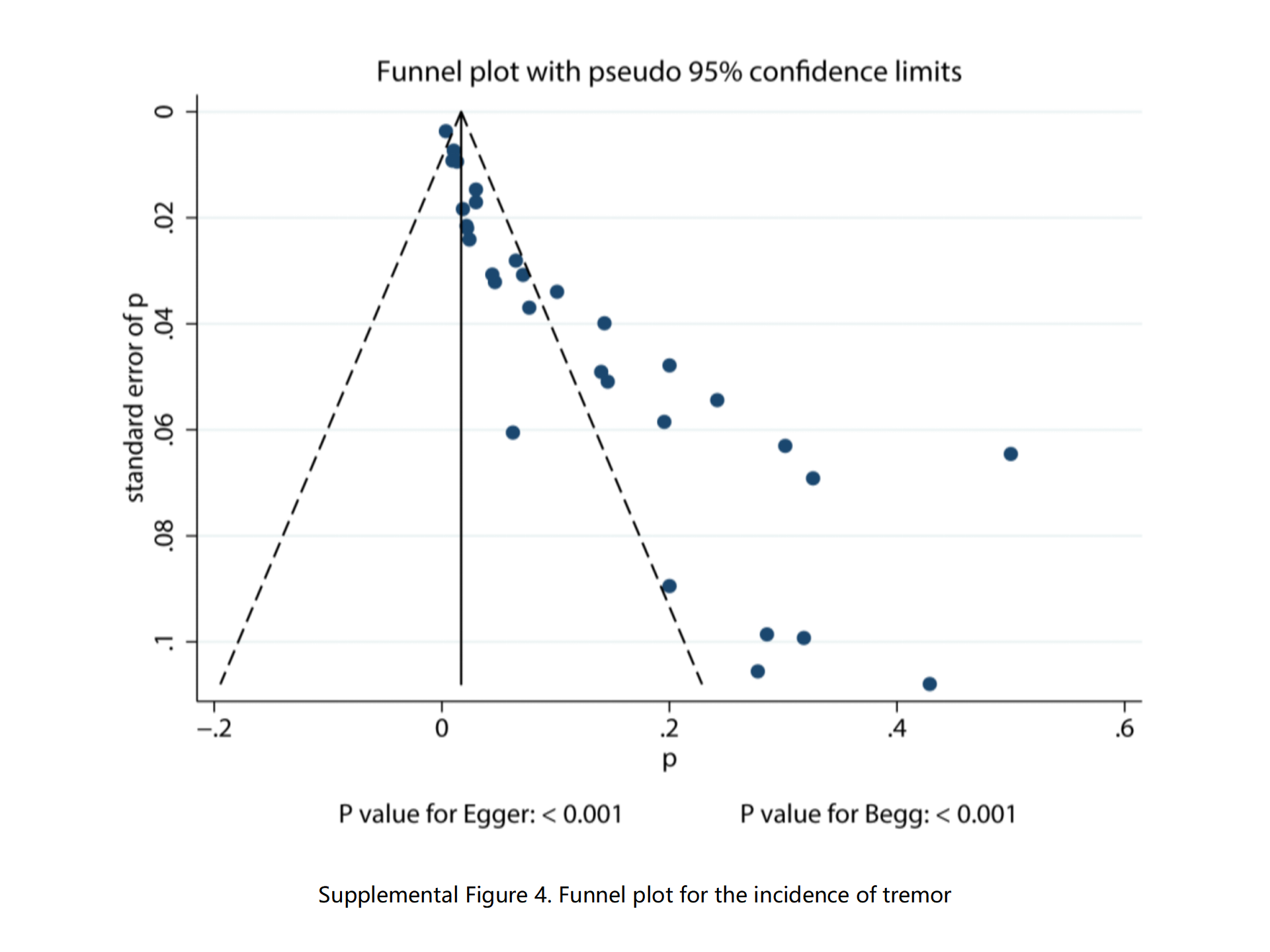

Supplement: Supplementary file 4 — Figure S4. Funnel plot for the incidence of tremor. [file CRJ-17-1254-s005.tif]

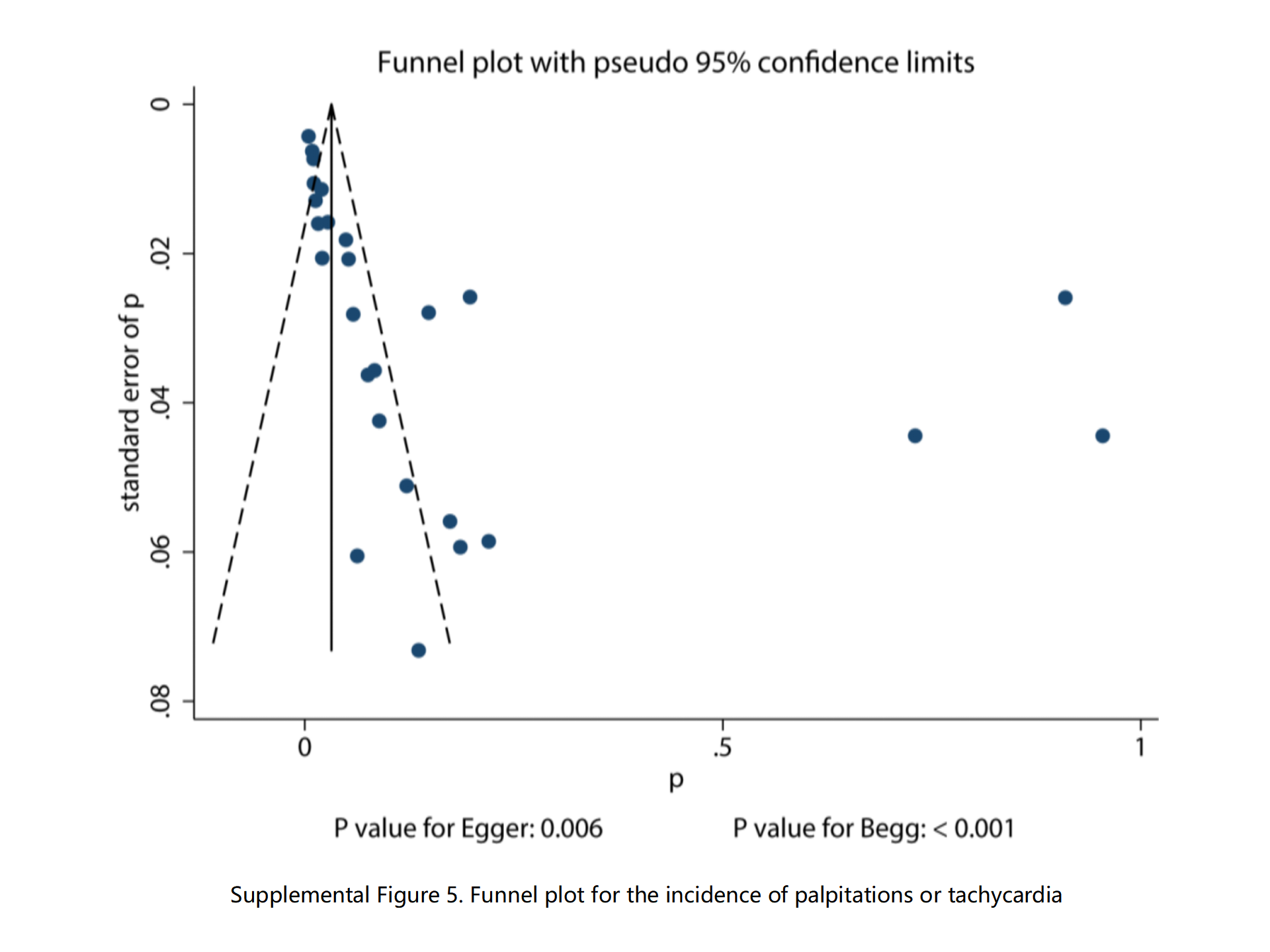

Supplement: Supplementary file 5 — Figure S5. Funnel plot for the incidence of palpitations or tachycardia. [file CRJ-17-1254-s015.tif]

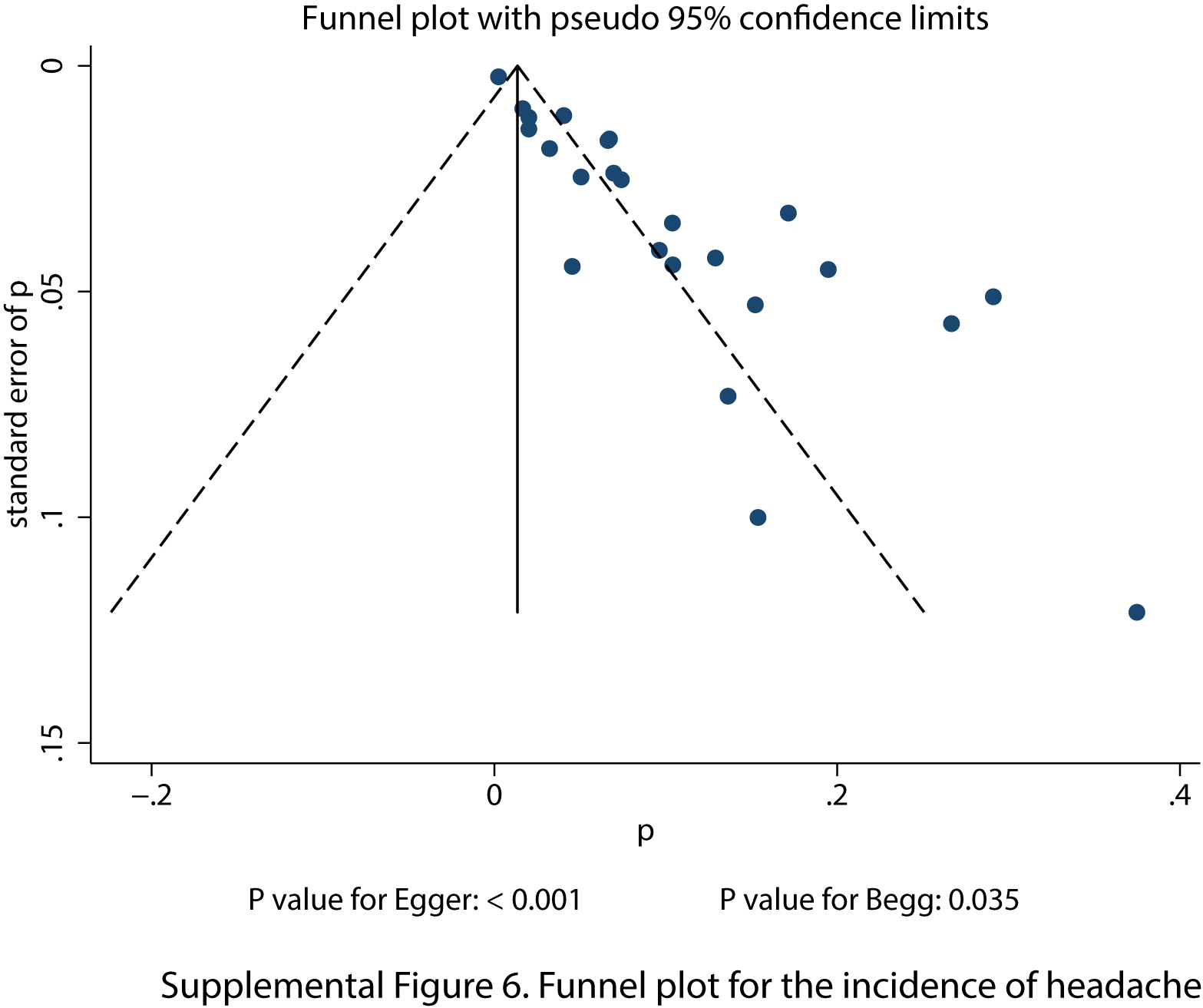

Supplement: Supplementary file 6 — Figure S6. Funnel plot for the incidence of headache. [file CRJ-17-1254-s018.tif]

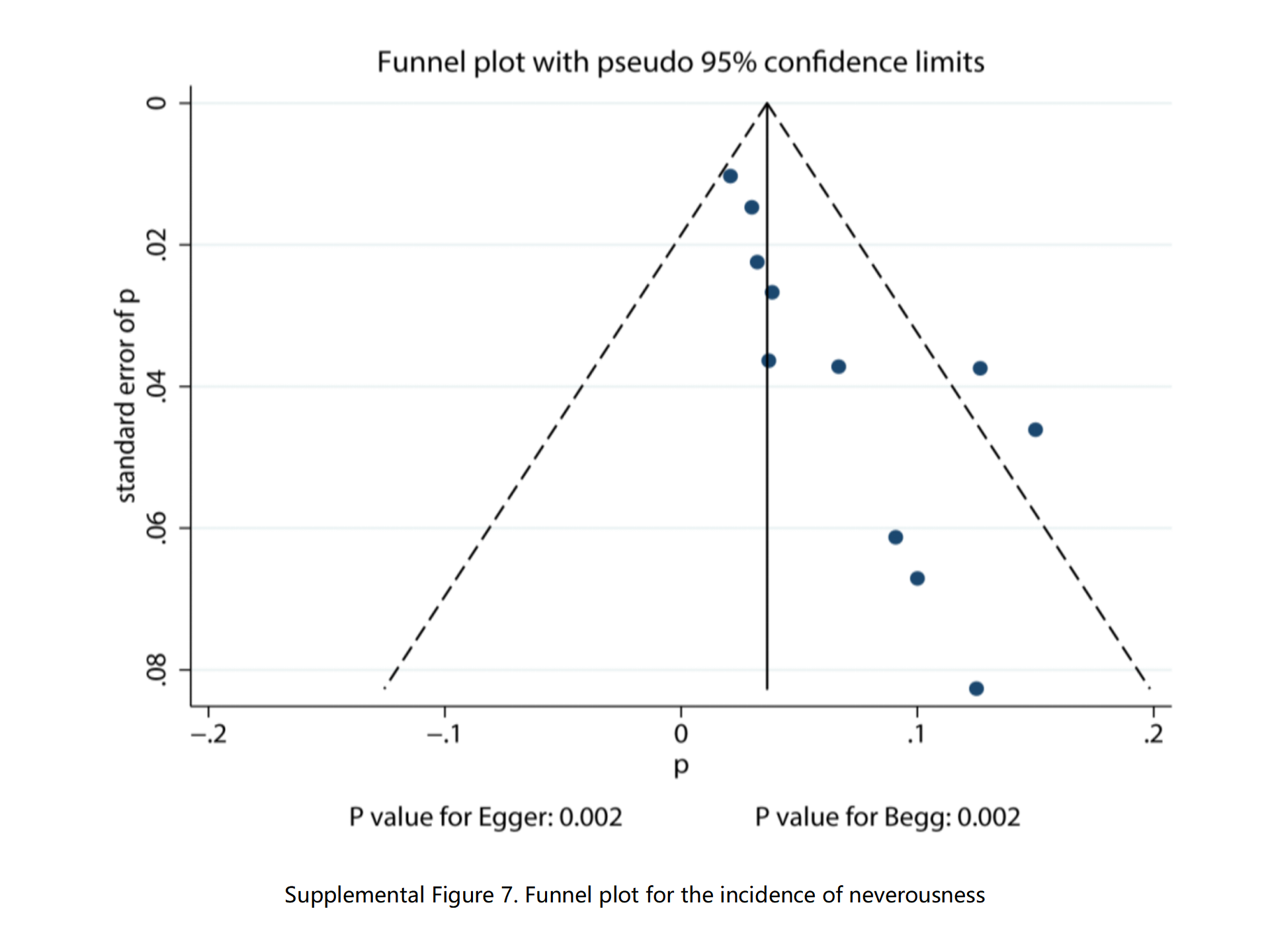

Supplement: Supplementary file 7 — Figure S7. Funnel plot for the incidence of nervousness. [file CRJ-17-1254-s025.tif]

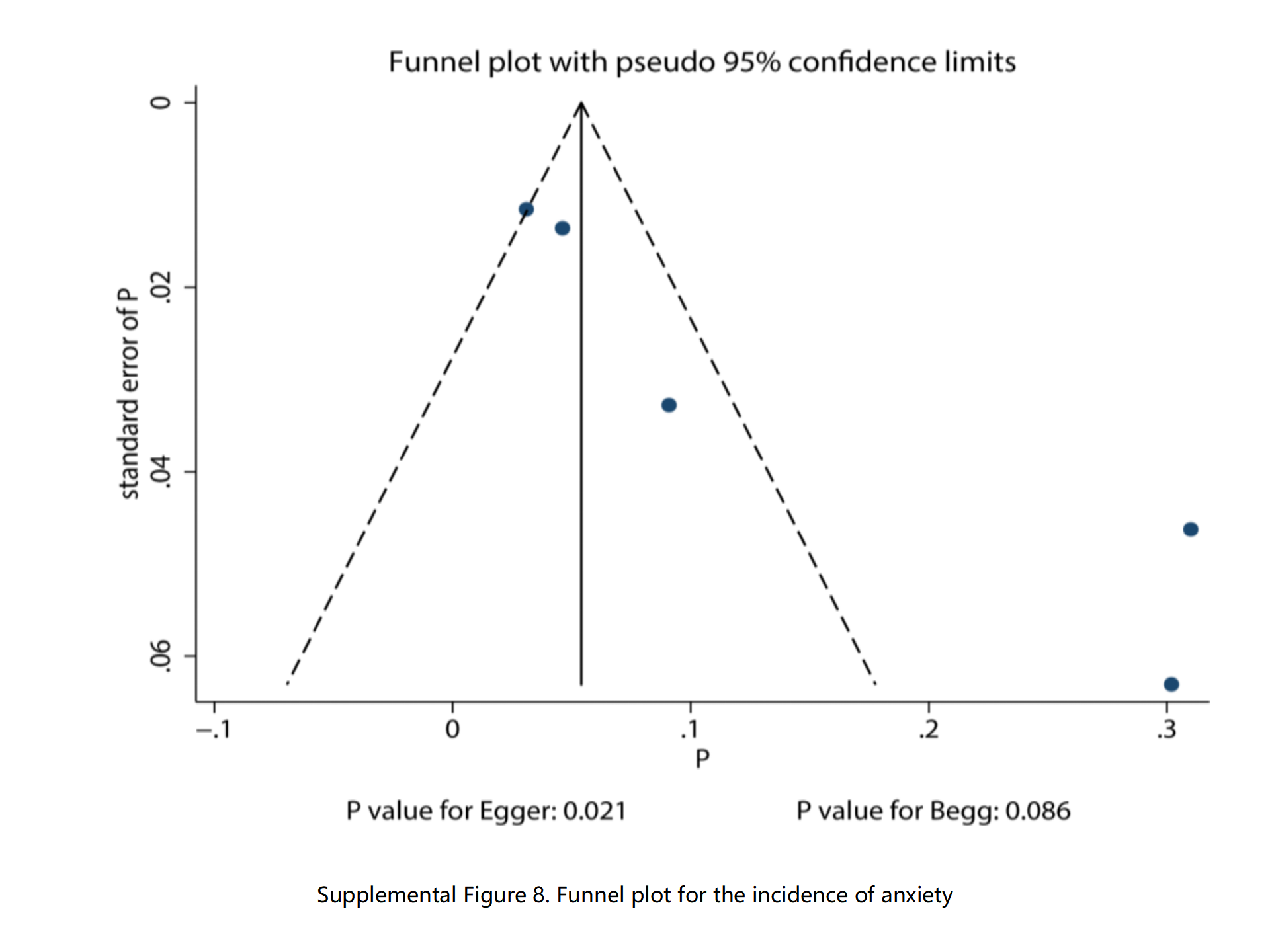

Supplement: Supplementary file 8 — Figure S8. Funnel plot for the incidence of anxiety. [file CRJ-17-1254-s010.tif]

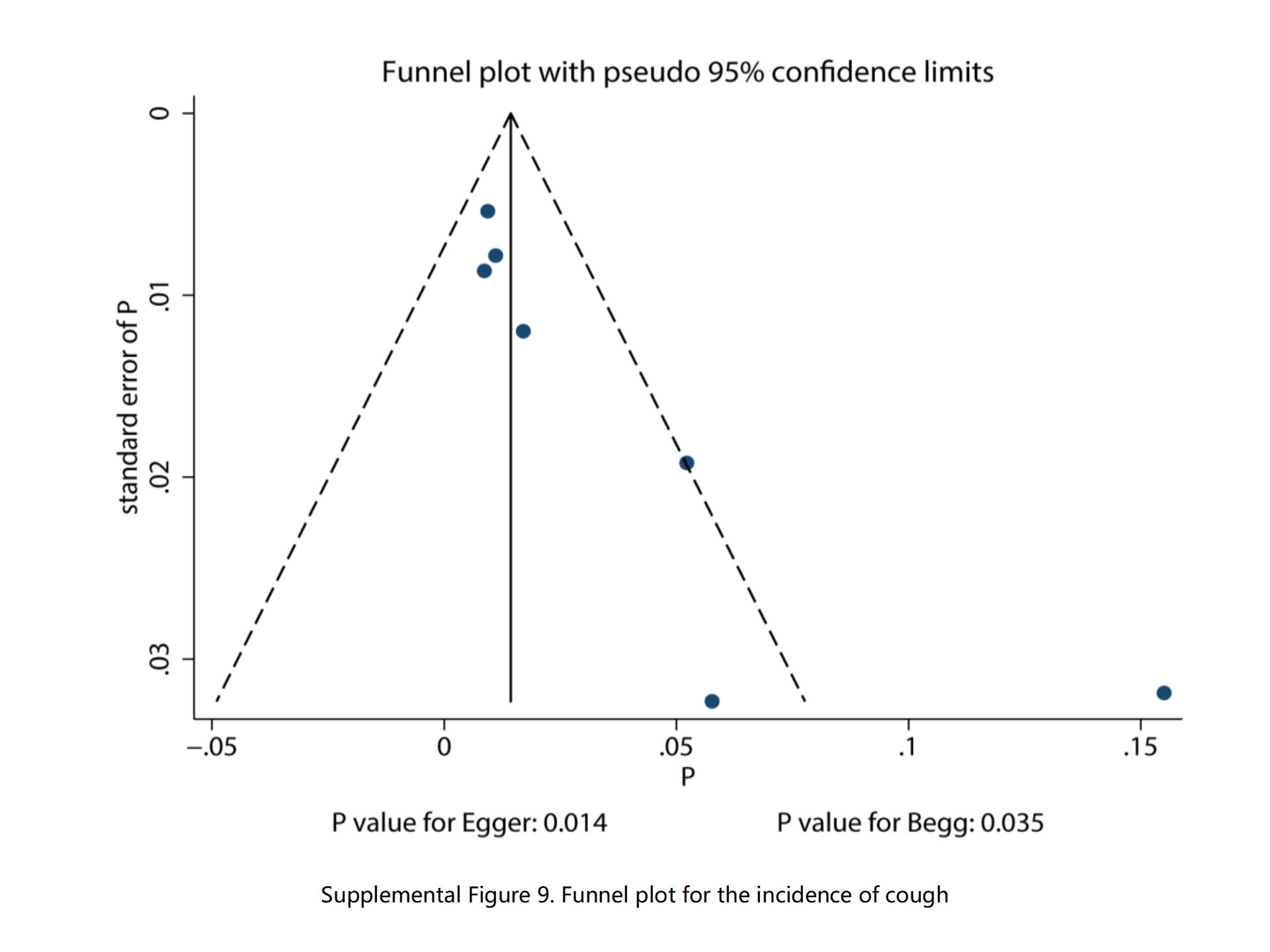

Supplement: Supplementary file 9 — Figure S9. Funnel plot for the incidence of cough. [file CRJ-17-1254-s003.tif]

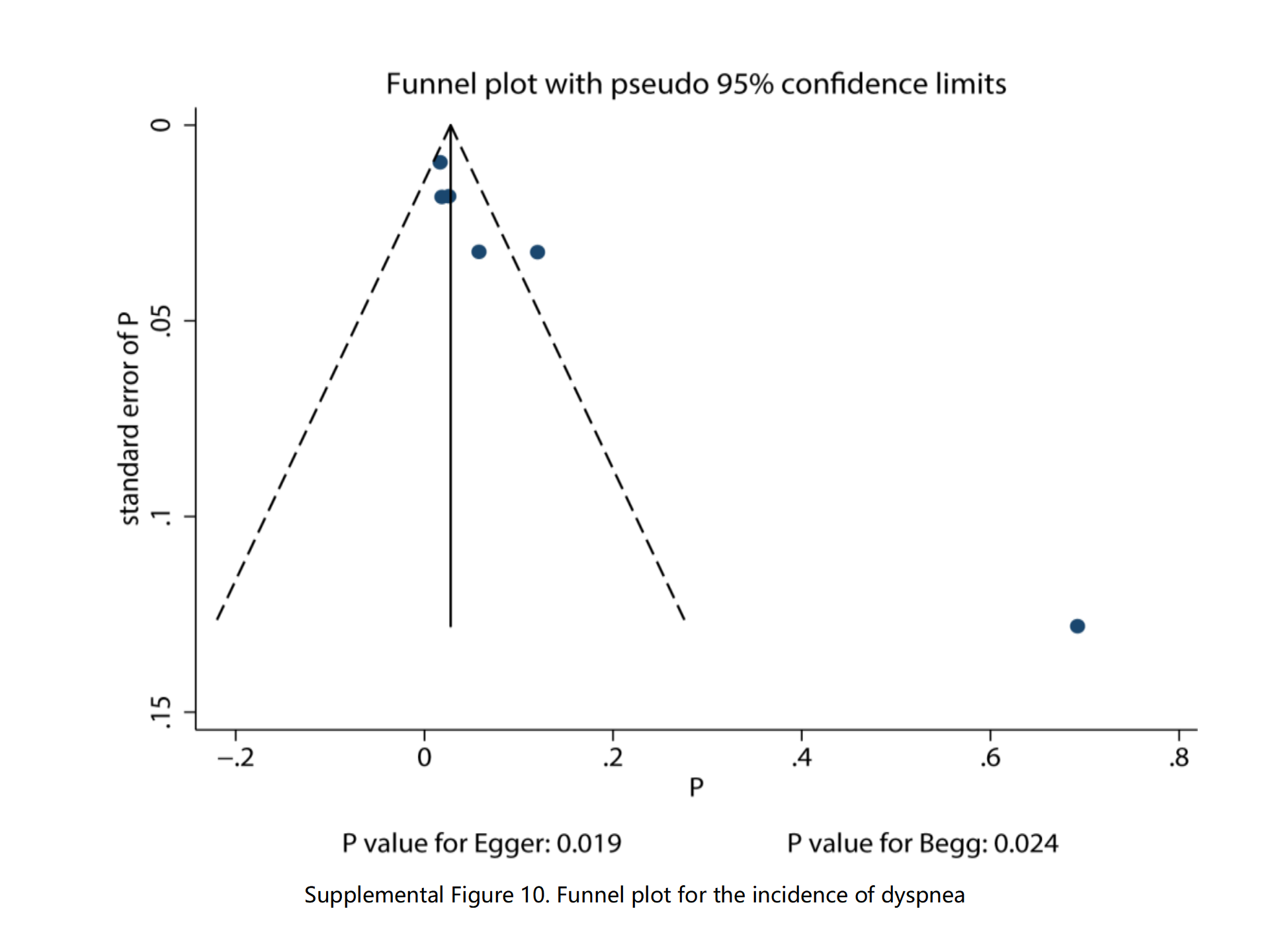

Supplement: Supplementary file 10 — Figure S10. Funnel plot for the incidence of dyspnoea. [file CRJ-17-1254-s012.tif]

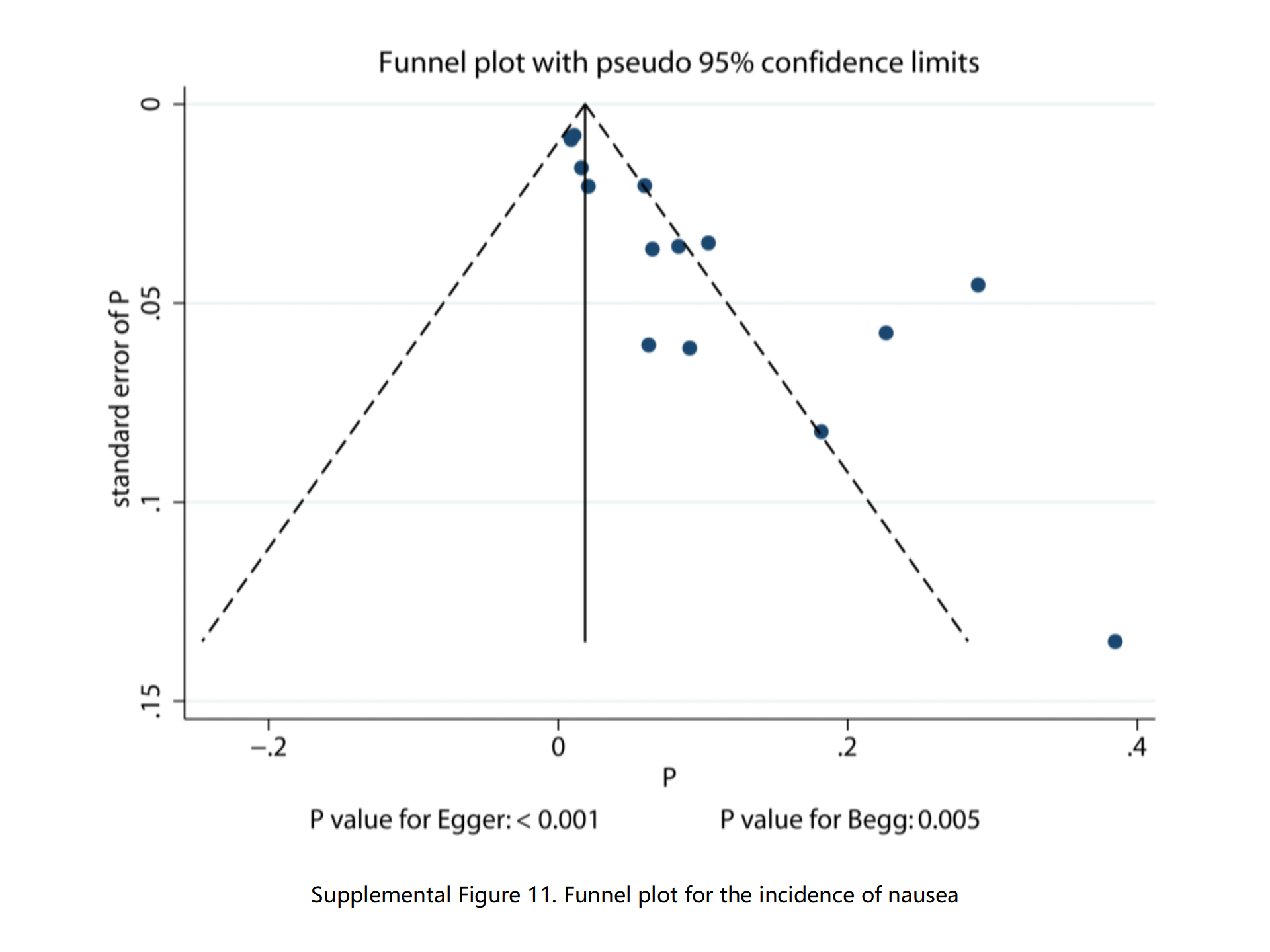

Supplement: Supplementary file 11 — Figure S11. Funnel plot for the incidence of nausea. [file CRJ-17-1254-s017.tif]

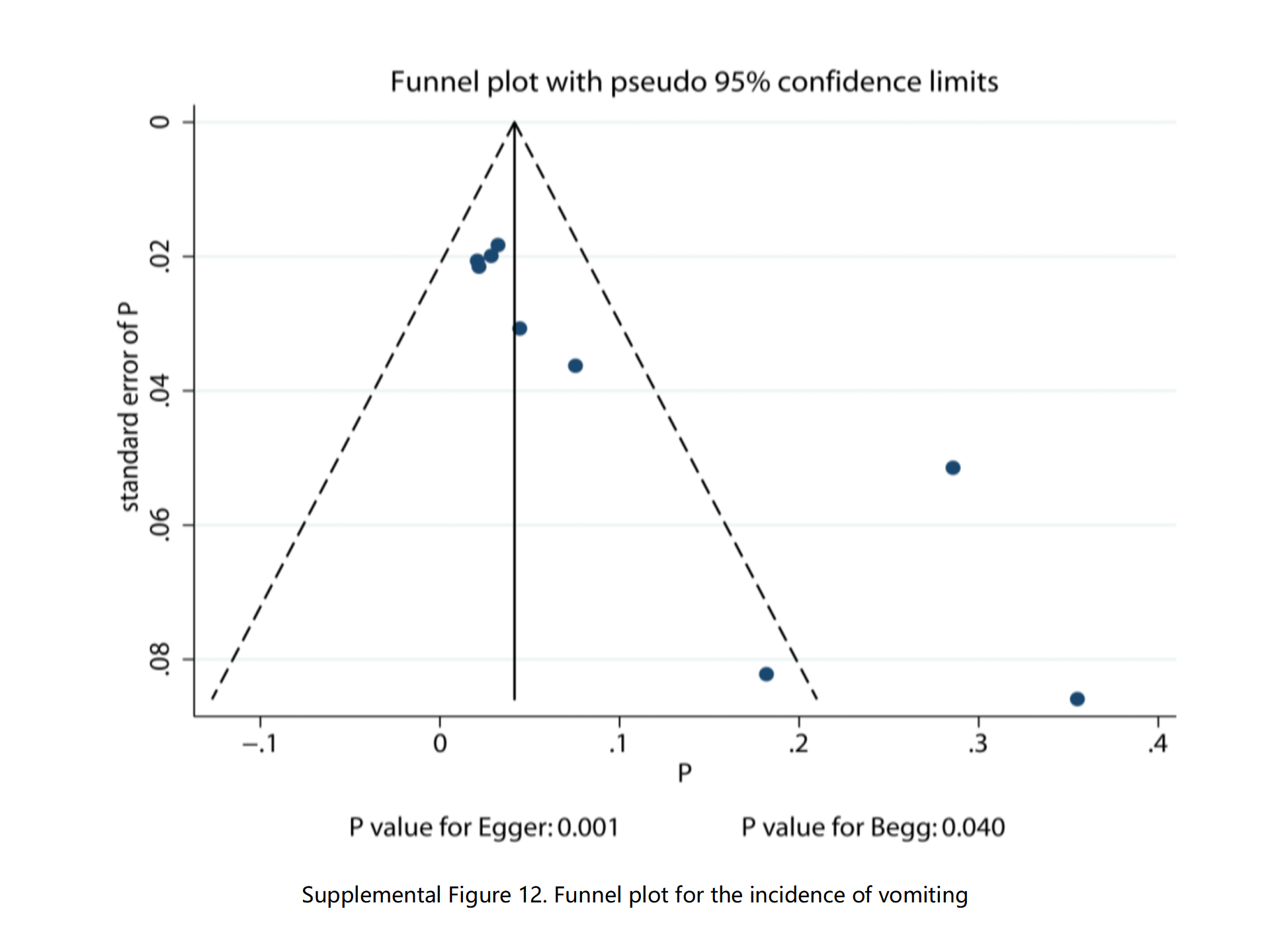

Supplement: Supplementary file 12 — Figure S12. Funnel plot for the incidence of vomiting. [file CRJ-17-1254-s008.tif]

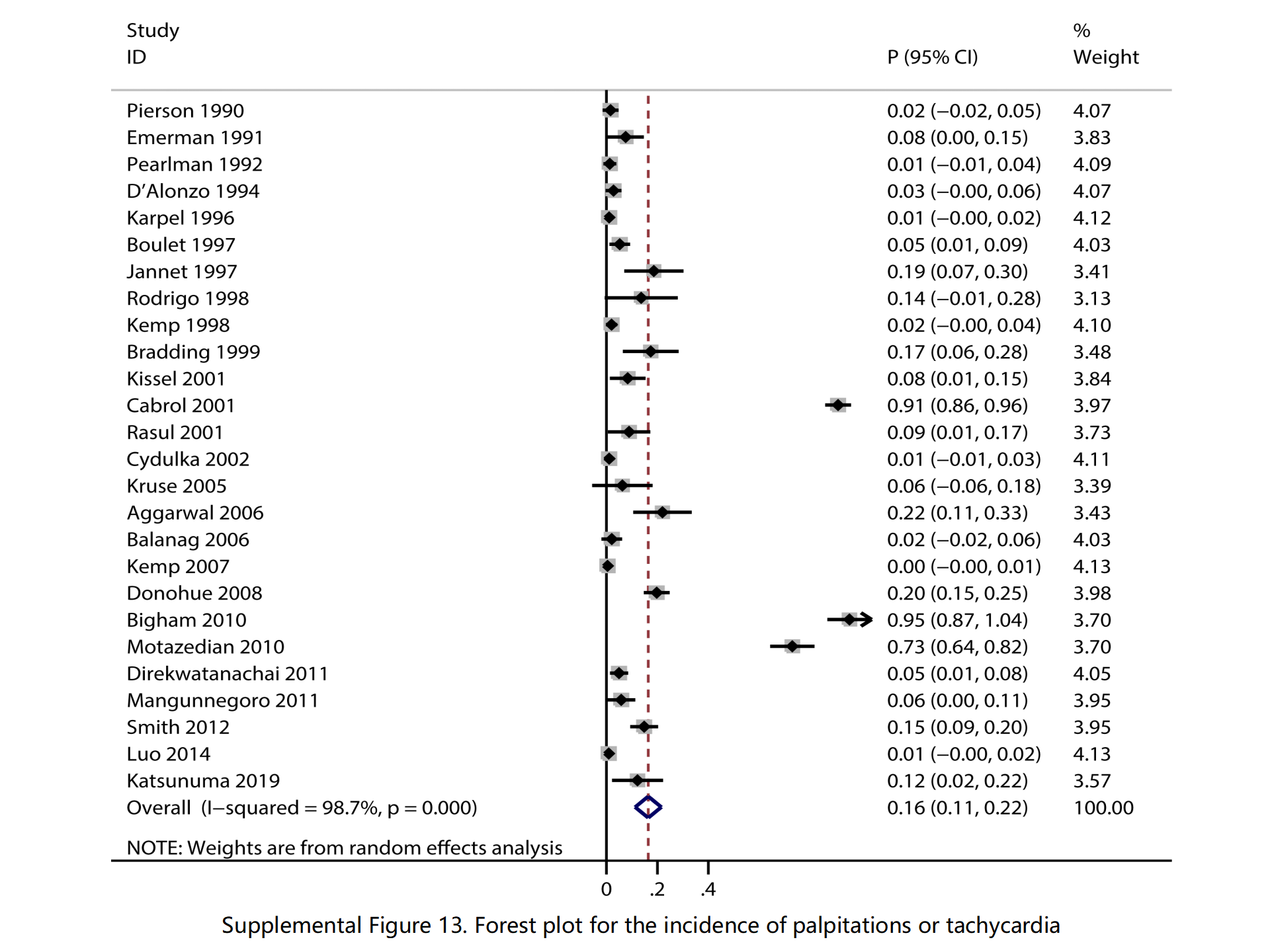

Supplement: Supplementary file 13 — Figure S13. Forest plot for the incidence of palpitations or tachycardia. [file CRJ-17-1254-s001.tif]

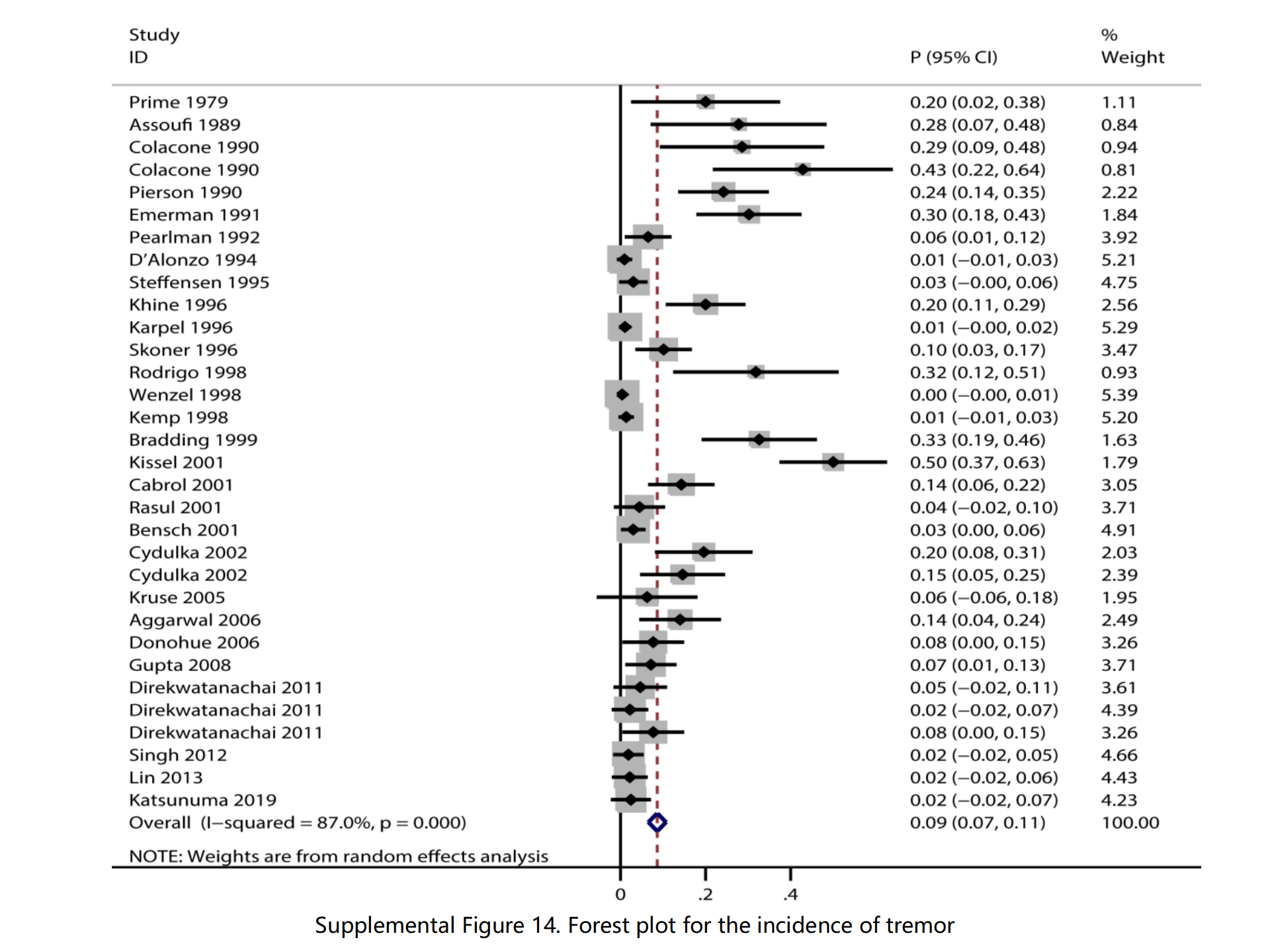

Supplement: Supplementary file 14 — Figure S14. Forest plot for the incidence of tremor. [file CRJ-17-1254-s022.tif]

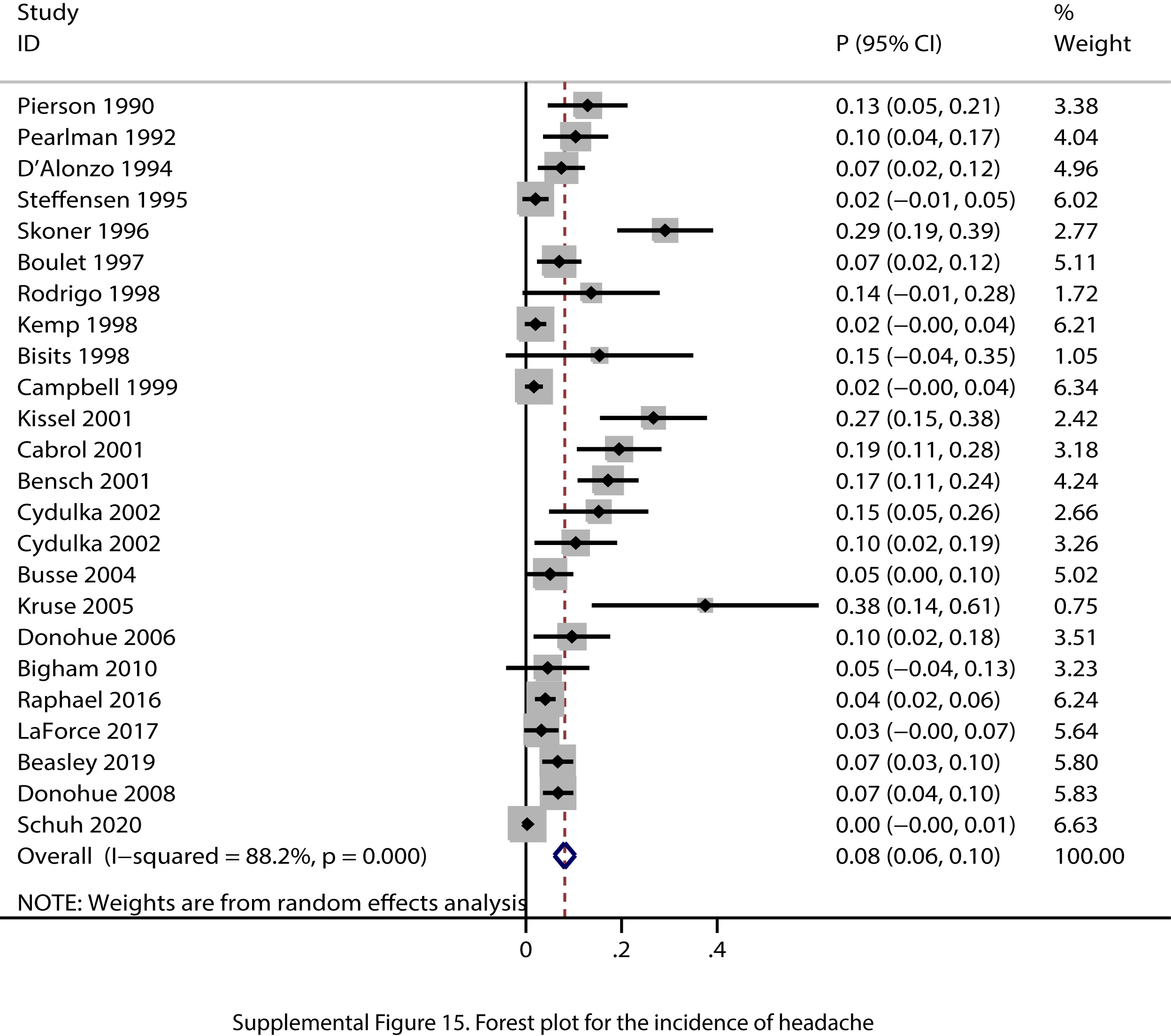

Supplement: Supplementary file 15 — Figure S15. Forest plot for the incidence of headache. [file CRJ-17-1254-s019.tif]

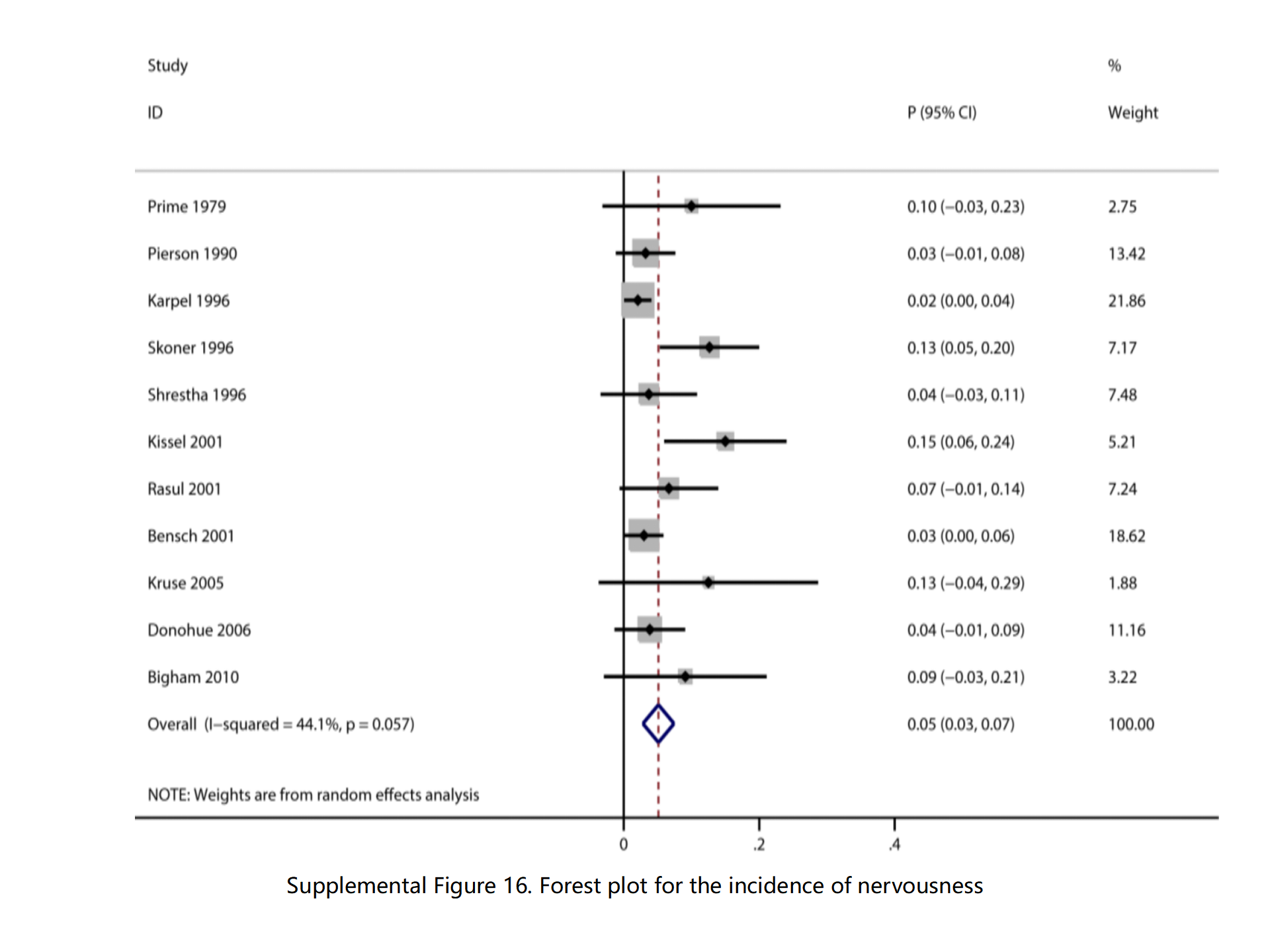

Supplement: Supplementary file 16 — Figure S16. Forest plot for the incidence of nervousness. [file CRJ-17-1254-s024.tif]

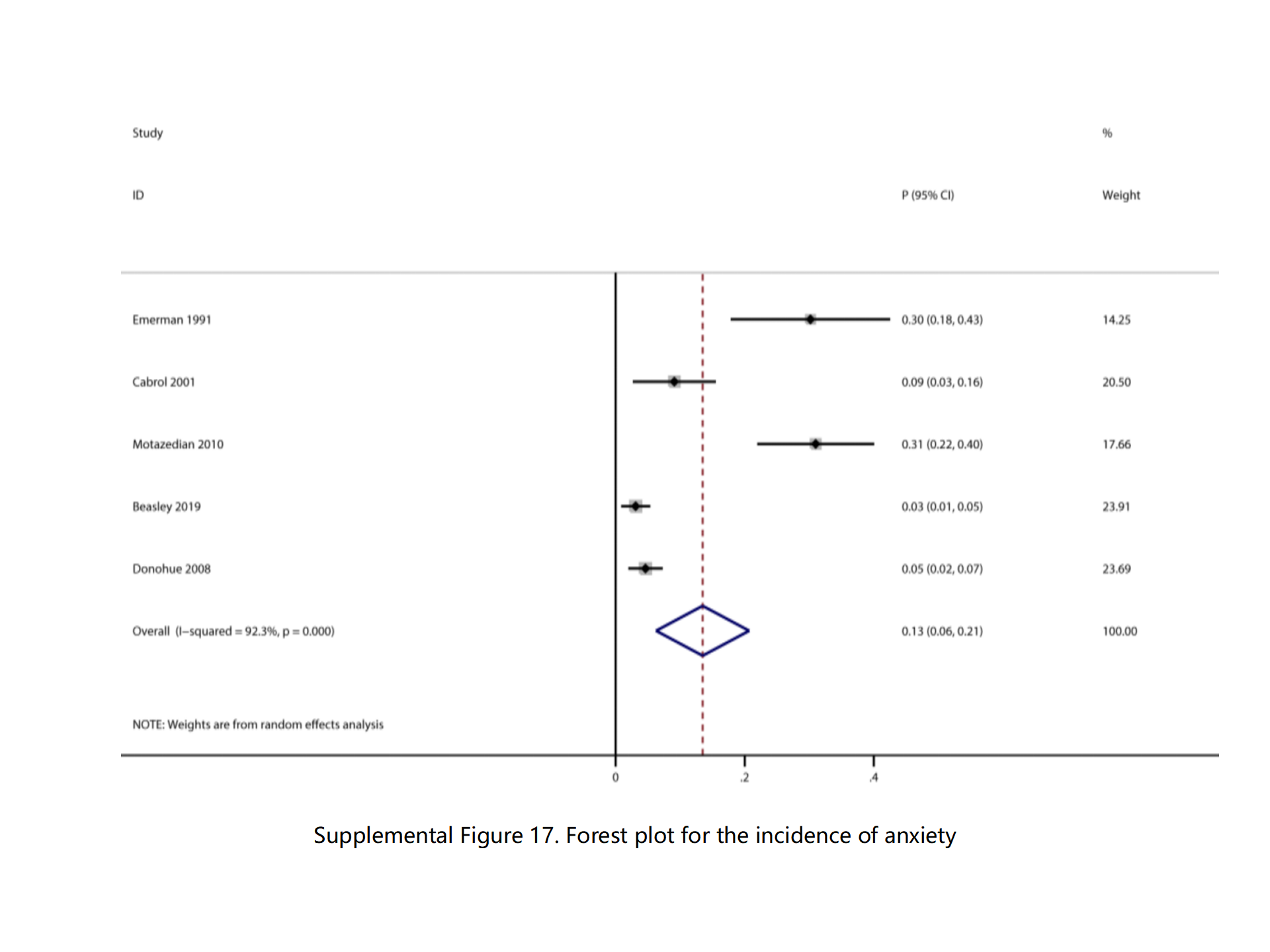

Supplement: Supplementary file 17 — Figure S17. Forest plot for the incidence of anxiety. [file CRJ-17-1254-s027.tif]

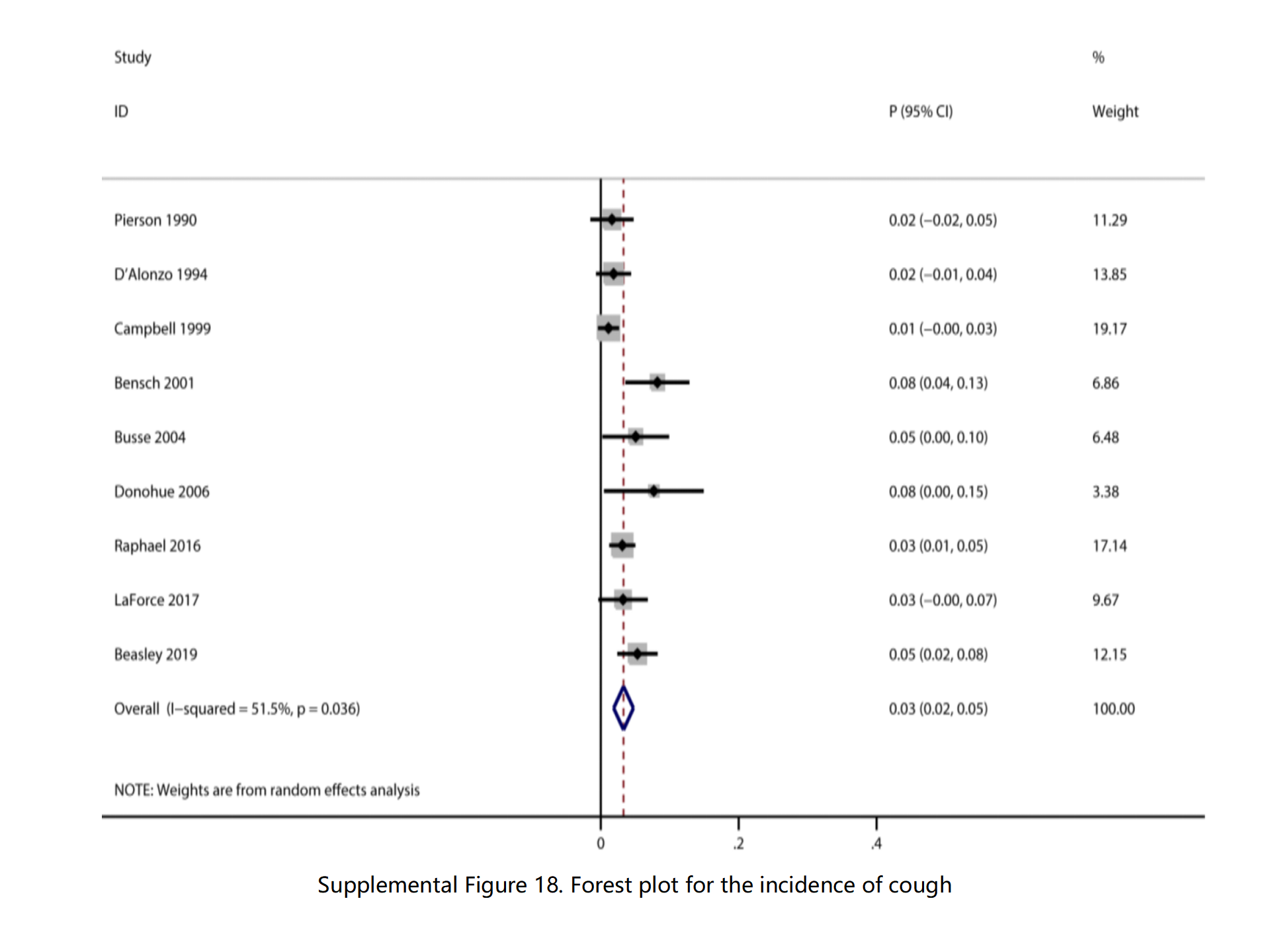

Supplement: Supplementary file 18 — Figure S18. Forest plot for the incidence of cough. [file CRJ-17-1254-s016.tif]

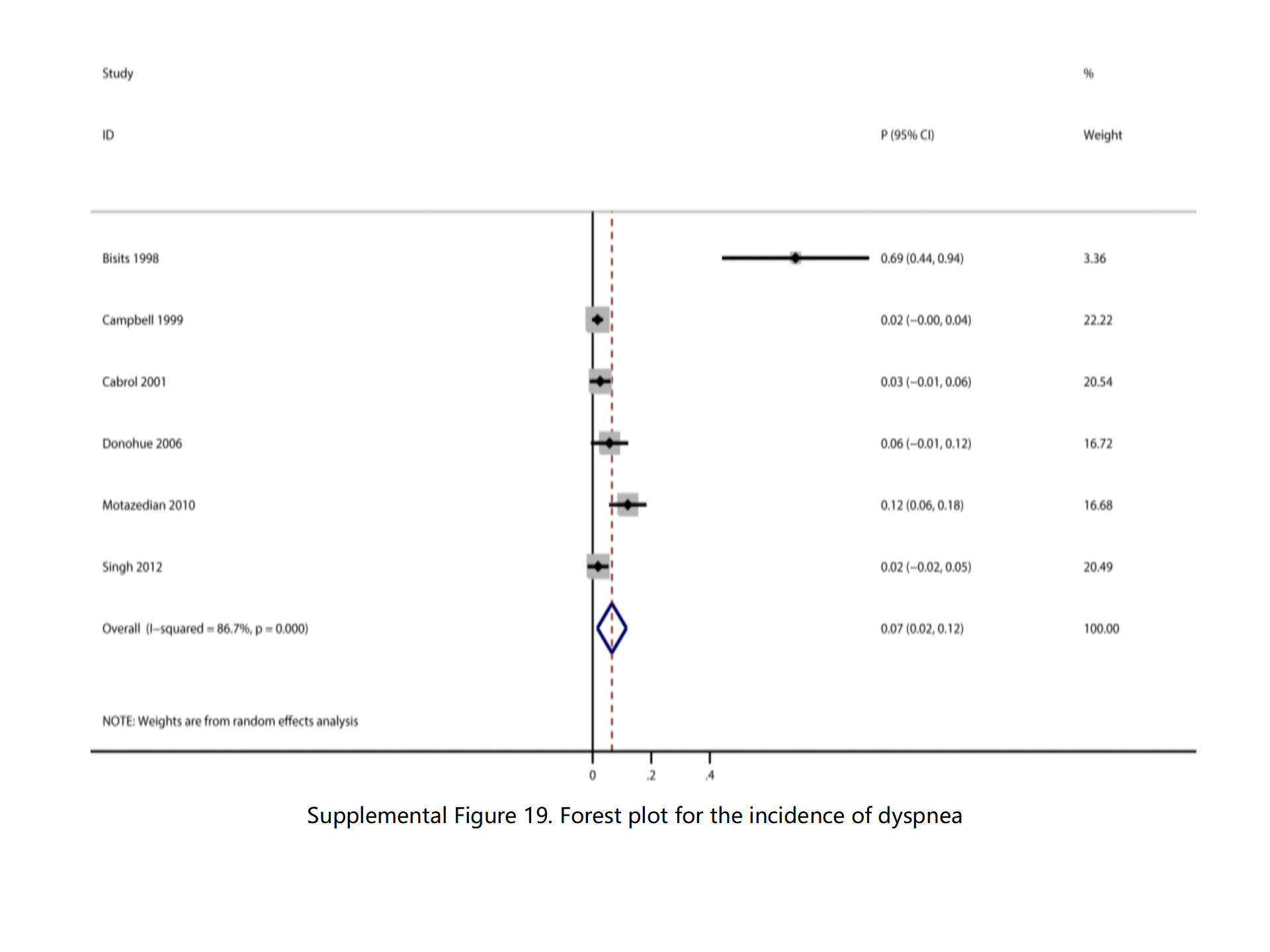

Supplement: Supplementary file 19 — Figure S19. Forest plot for the incidence of dyspnoea. [file CRJ-17-1254-s011.tif]

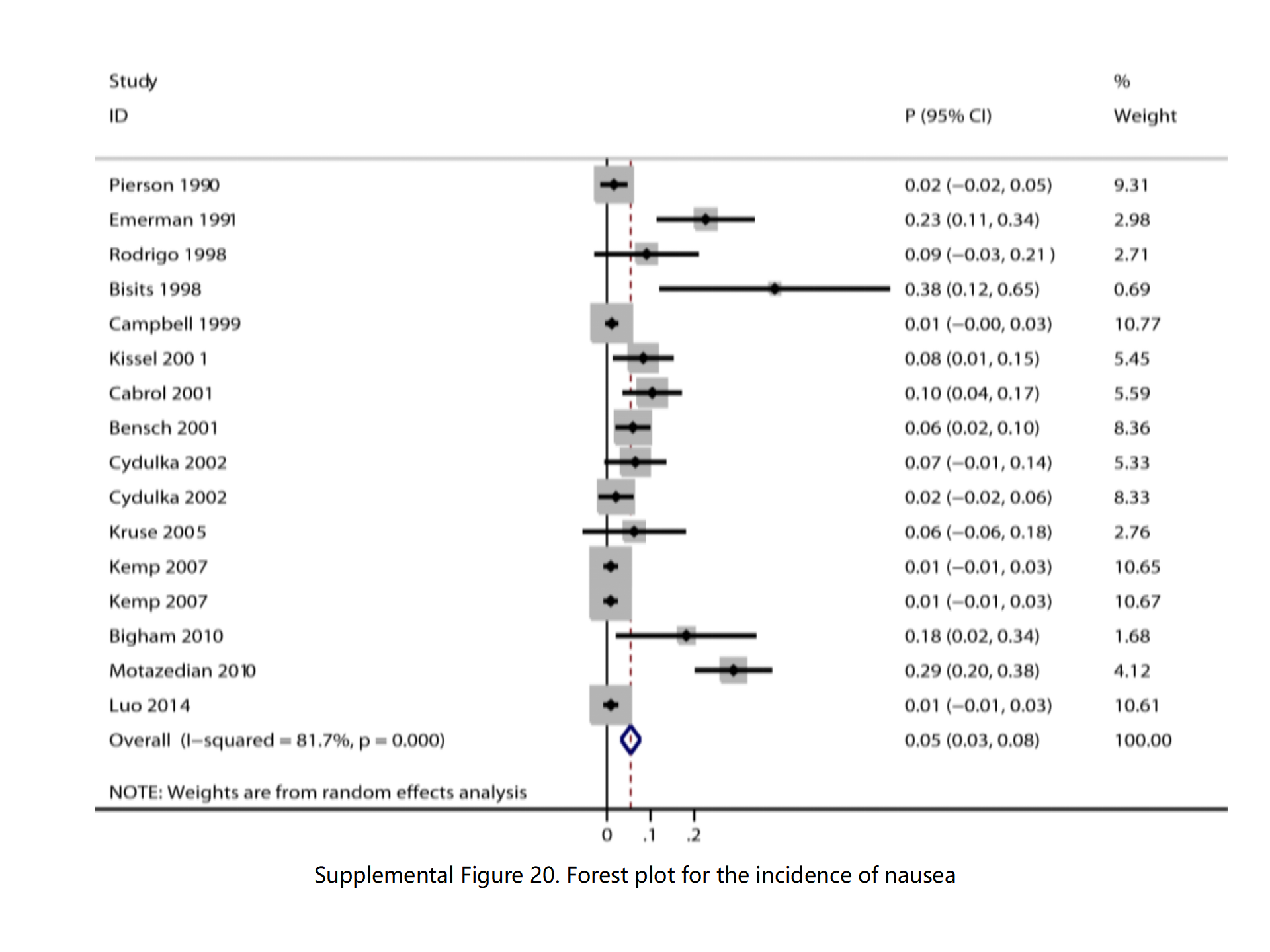

Supplement: Supplementary file 20 — Figure S20. Forest plot for the incidence of nausea. [file CRJ-17-1254-s014.tif]

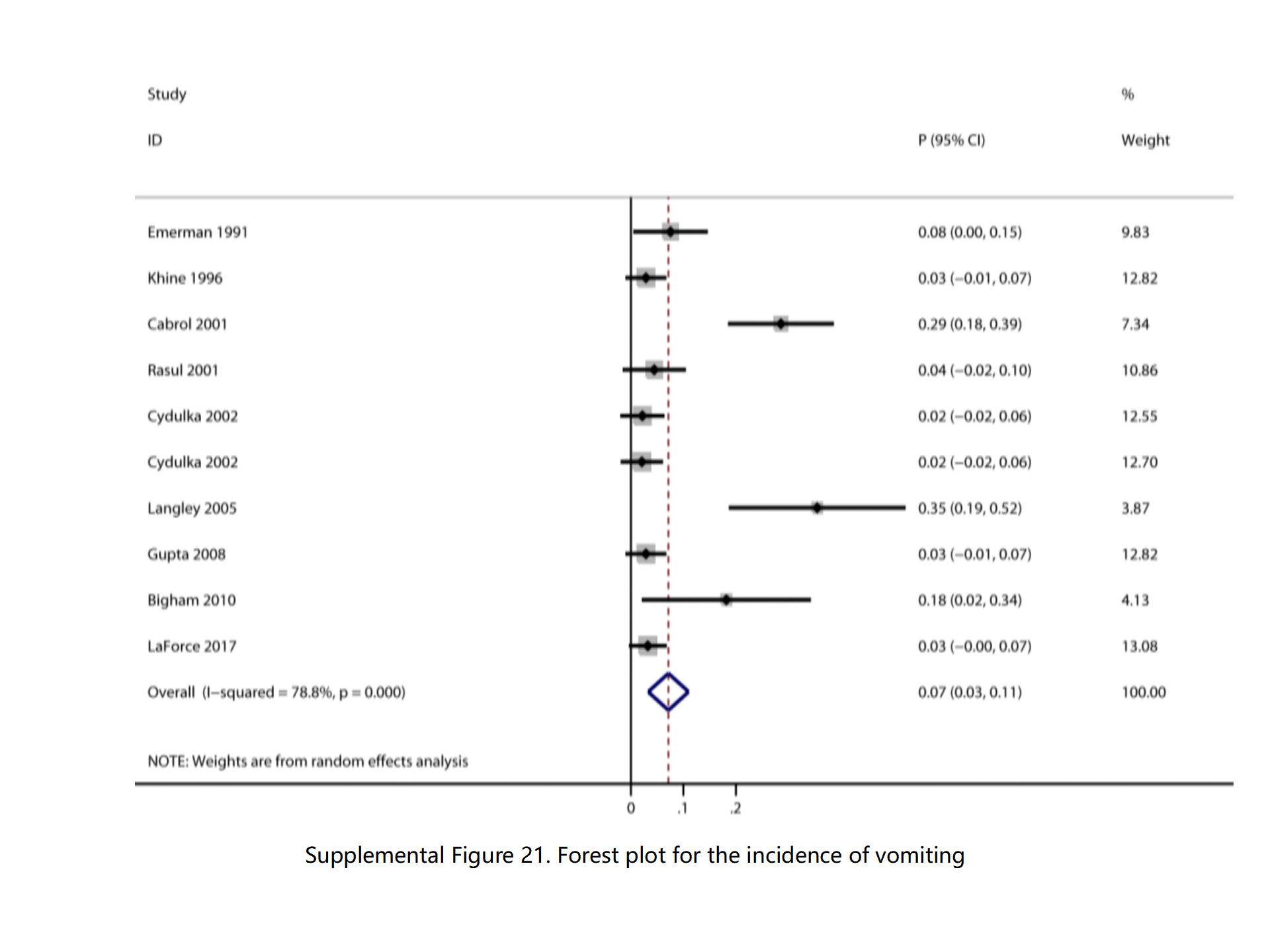

Supplement: Supplementary file 21 — Figure S21. Forest plot for the incidence of vomiting. [file CRJ-17-1254-s013.tif]

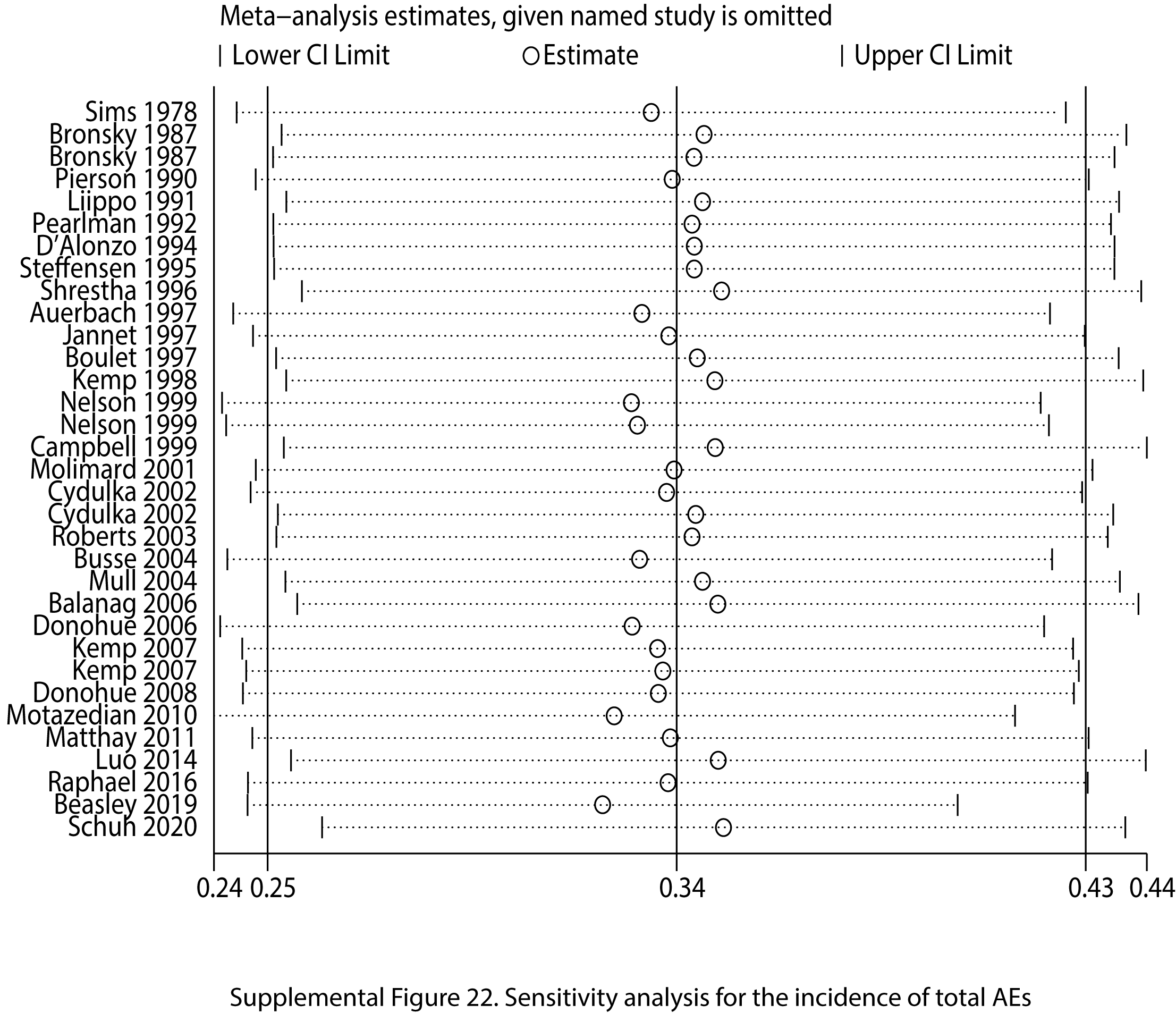

Supplement: Supplementary file 22 — Figure S22. Sensitivity analysis for the incidence of total AEs. [file CRJ-17-1254-s004.tif]

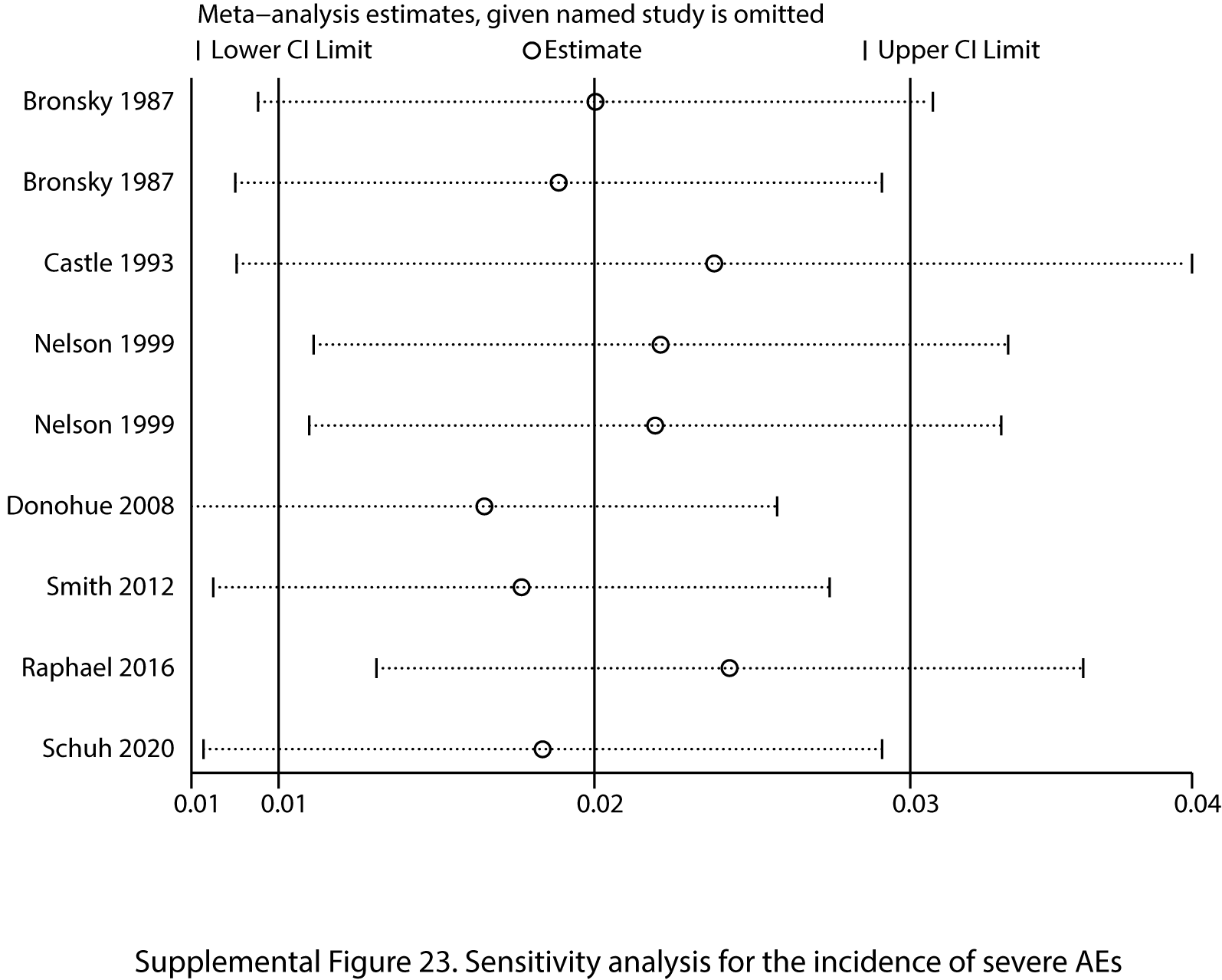

Supplement: Supplementary file 23 — Figure S23. Sensitivity analysis for the incidence of severe AEs. [file CRJ-17-1254-s006.tif]

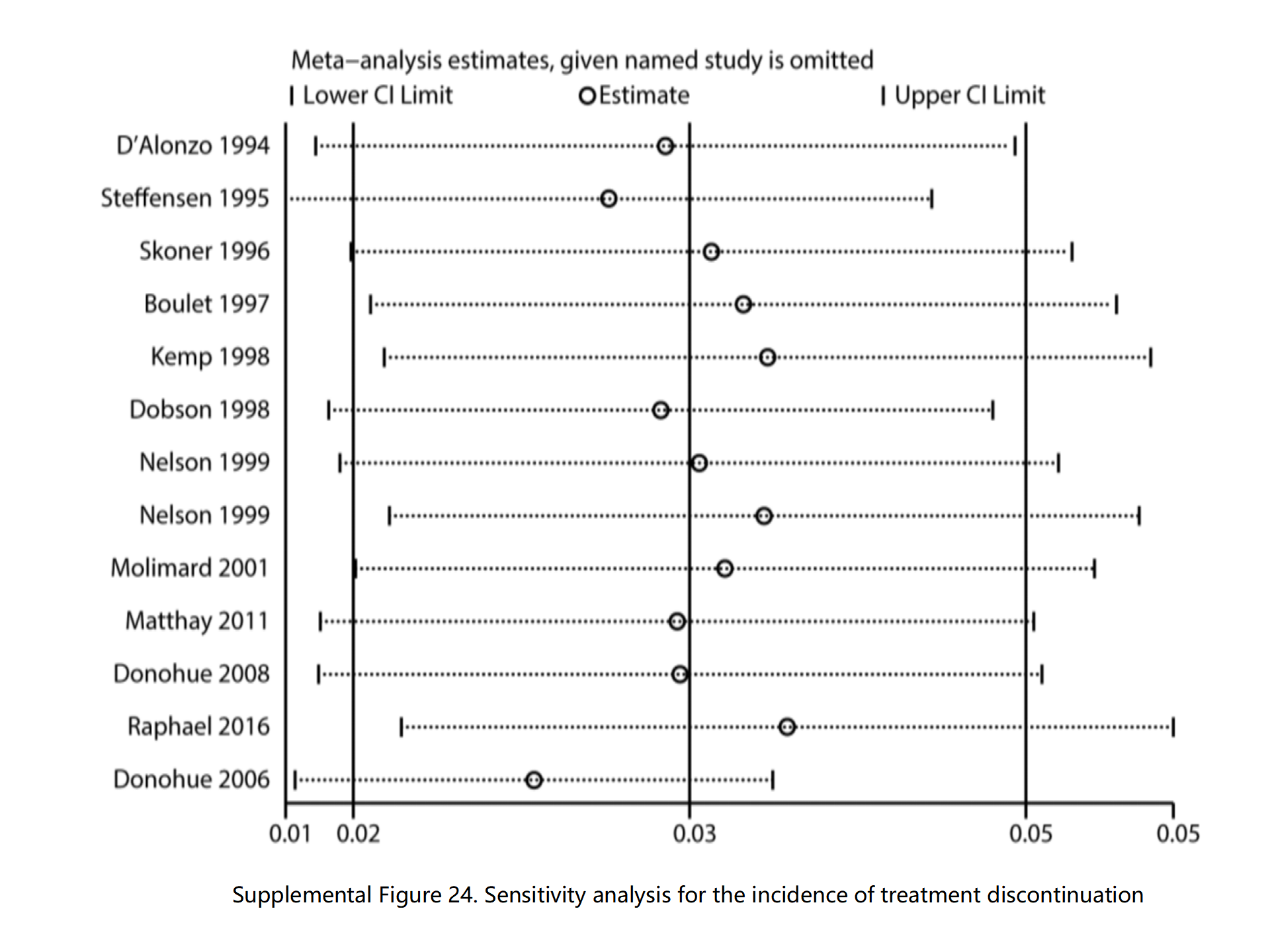

Supplement: Supplementary file 24 — Figure S24. Sensitivity analysis for the incidence of treatment discontinuation. [file CRJ-17-1254-s021.tif]
