# Supplementary material for: Safety outcomes of salbutamol: A systematic review and meta‐analysis
Source: Clin Respir J. 2023 Oct 16;17(12):1254–64. doi: 10.1111/crj.13711 (PMC10730473; doi:10.1111/crj.13711)
Supplement: Supplementary file 25 — Data S1. Supporting Information. [file CRJ-17-1254-s020.docx]

**Supplemental Material 1 Literature search strategy**

**PubMed:**

(salbutamol OR albuterol OR Albuterol[Mesh]) AND (((((("randomized controlled trial"[Publication Type]) OR "controlled clinical trial"[Publication Type]) OR (randomized OR randomised OR randomly OR groups OR trial OR placebo)) OR drug therapy[MeSH Terms]))) NOT ((animals [mh] NOT humans [mh]))

**EmBase:**

('salbutamol OR 'salbutamol'/exp) AND (randomized OR randomised OR randomly OR groups OR trial OR placebo OR 'drug therapy' OR 'randomized controlled trial topic'/exp OR 'randomized controlled trial'/exp OR 'controlled clinical trial'/exp OR ((single:ab,ti OR doubl*:ab,ti OR tripl*:ab,ti OR treb*:ab,ti) AND (blind*:ab,ti OR mask*:ab,ti)) NOT (animals NOT humans))

**Cochrane library:**

MESH DESCRIPTOR salbutamol OR albuterol AND (randomized controlled trial OR controlled clinical trial OR randomized OR randomised OR randomly OR trial OR groups NOT (animals NOT humans))
