# Supplementary material for: Safety outcomes of salbutamol: A systematic review and meta‐analysis
Source: Clin Respir J. 2023 Oct 16;17(12):1254–64. doi: 10.1111/crj.13711 (PMC10730473; doi:10.1111/crj.13711)
Supplement: Supplementary file 26 — Table S1. The baseline characteristics of identified trials and patients. [file CRJ-17-1254-s007.docx]

Supplemental Table 1. The baseline characteristics of identified trials and patients

| Study ID | Publication year | Country | No. of patients | Mean age years (range) | Indication | Treatment | Formulation | Outcomes of AEs |
| --- | --- | --- | --- | --- | --- | --- | --- | --- |
| Sims 1978 ^24^ | 1978 | UK | 42 | NR | Preterm labour | Salbutamol 5pg/min for 24 hour | Intravenous | Total AEs |
| Prime 1979 ^25^ | 1979 | UK | NR | 41.2 (20~62) | COPD | Salbutamol 4mg/day for 3 days | Oral | Tremor, nervousness |
| Bronsky 1987 ^26^ | 1987 | USA | 115  116 | 33.7 (12~76)  33.2 (12~67) | Asthma | Arm 1: salbutamol powder: 200 μg/day for 1 day; Arm 2: albuterol aerosol (two puffs, I80 μg) | Inhaled | Total AEs, severe AEs |
| Assoufi 1989 ^27^ | 1989 | UK | 18 | 64.4 (36~80) | COPD | Salbutamol 1 mg, 2 mg and 5 mg daily for 6 days | Inhaled | Tremor |
| Colacone 1990 ^28^ | 1990 | Canada | 21  21 | NR | Asthma | Arm 1: salbutamol bolus nebulization, 10mg/day for 1 day; Arm 2: salbutamol continuous nebulization, 10mg/day for 1 day | Inhaled | Tremor |
| Pierson 1990 ^29^ | 1990 | USA | 62 | 28 (12~67) | Asthma | Salbutamol 16mg/day for 12 weeks | Oral | Total AEs, tremor, headache, nervousness, nausea, cough, palpitations or tachycardia |
| Emerman 1991 ^30^ | 1991 | USA | 53 | 31.4 | Asthma | 0.5% salbutamol, three doses for 1 day | Inhaled | Tremor, anxiety, nausea, vomiting, palpitations or tachycardia |
| Liippo 1991 ^31^ | 1991 | Finland | 20 | 44 | Asthma | Two puffs salbutamol (200 μg) for 1 day | Inhaled | Total AEs |
| Pearlman 1992 ^32^ | 1992 | USA | 77 | 28 | Asthma | Salbutamol (180 μg four times a day) for 12 weeks | Inhaled | Total AEs, headache, tremor, palpitations or tachycardia |
| Castle 1993 ^33^ | 1993 | UK | 8393 | >12 | Asthma | Salbutamol (200 μg four times a day) for 16 weeks | Inhaled | Severe AEs |
| D'Alonzo 1994 ^34^ | 1994 | USA | 108 | 31 | Asthma | Salbutamol (180 μg four times a day) for 12 weeks | Inhaled | Total AEs, treatment discontinuation, headache, cough, tremor, palpitations or tachycardia |
| Steffensen 1995 ^35^ | 1995 | Sweden | 100 | 48 | COPD | salbutamol (400 μg four times a day) for 12 weeks for 12 weeks | Inhaled | Total AEs, treatment discontinuation, tremor, headache |
| Karpel 1996 ^36^ | 1996 | USA | 192 | 32.5 | Asthma | 2.5 mg of salbutamol twice a day for 1day | Inhaled | Tremor, nervousness, palpitations or tachycardia |
| Khine 1996 ^37^ | 1996 | USA | 35  35 | 8.1  7.2 | Asthma | Arm 1: salbutamol intermittent nebulized (0.15 mg/kg/dose every 30 min); Arm 2: salbutamol continuous nebulization (0.3 mg/kg/ hr) for 2 hours | Inhaled | Vomiting, tremor |
| Shrestha 1996 ^38^ | 1996 | USA | 27 | 35 | Asthma | 2.5 mg of salbutamol four times a day for 2 weeks | Inhaled | Total AEs, nervousness |
| Skoner 1996 ^39^ | 1996 | USA | 79 | 10 | Asthma | Salbutamol 4 mg twice daily, increasing up to 12 mg q 12h for 4 weeks. | Oral | Tremor, treatment discontinuation, headache, nervousness |
| Auerbach 1997 ^40^ | 1997 | UK | 216 | 64.6 (41~85) | COPD | Salbutamol sulfate inhalation solution (3.0 mg) for 85 days. | Inhaled | Total AEs |
| Boulet 1997 ^41^ | 1997 | Canada | 115 | 40 | Asthma | Salbutamol 200 μg four times daily for 12 weeks. | Inhaled | Total AEs, headache, palpitations or tachycardia, treatment discontinuation |
| Jannet 1997 ^42^ | 1997 | France | 43 | 28.9 | Preterm labour | Salbutamol 2.5 mg in a 500 ml 5% weight per volume glucose solution, with an initial flow-rate of 30 ml/h, 0.15 mg/h salbutamol for 2 hours. | Intravenous | Total AEs, palpitations or tachycardia |
| Bisits 1998 ^43^ | 1998 | Australia | 13 | 23.6 | Preterm labour | Continuous salbutamol treatment at an initial infusion rate of 25 μg/min | Intravenous | Dyspnea, nausea, headache |
| Dobson 1998 ^44^ | 1998 | USA | 23 | 5.16 month | Bronchiolitis | Salbutamol 1.25 mg for patients < 10 kg, 2.5 mg for patients > 10 kg, every 2 hours for the first 24 hours, then every 4 hours for the next 48 hours | Inhaled | Treatment discontinuation |
| Kemp 1998 ^45^ | 1998 | USA | 150 | 31 | Asthma | Salbutamol 180 μg four times daily for 12 weeks | Inhaled | Total AEs, headache, tremor, palpitations or tachycardia, treatment discontinuation. |
| Rodrigo 1998 ^46^ | 1998 | Uruguay | 11  11 | 33.1  31.7 | Asthma | Arm 1: salbutamol (MDl) four puffs at 10-minute intervals (2.4 mg/h); Arm 2: nebulized salbutamol 1.5 mg, via nebulizer at 15-minute intervals (6 mg/h) for 3 hours. | Inhaled | Tremor, headache, nausea, palpitations or tachycardia |
| Wenzel 1998 ^47^ | 1998 | USA | 275 | 33.8 (12~83) | Asthma | Salbutamol 180 μg four times daily for 12 weeks | Inhaled | Tremor |
| Bradding 1999 ^48^ | 1999 | UK | 24  22 | 32 (18~54)  28 (17~57) | Asthma | Arm 1: salbutamol four hourly nebulized 5mg for 4.7 days; Arm 2: salbutamol as required 2.5±5.0 mg for 3.7 days | Inhaled | Tremor, palpitations or tachycardia |
| Campbell 1999 ^49^ | 1999 | USA | 180 | NR | COPD | 180 μg of salbutamol four times daily for 29 days. | Inhaled | Total AEs, headache, cough, dyspnea, nausea |
| Nelson 1999 ^50^ | 1999 | USA | 97  92 | 34.2  34.6 | Asthma | Arm 1: salbutamol 108 μg/actuation (DPI), arm 2: salbutamol 90 μg/actuation (MDI) | Inhaled | Total AEs, severe AEs, treatment discontinuation |
| Bensch 2001 ^51^ | 2001 | USA | 134 | 35.7 | Asthma | Salbutamol, 180 μg four times daily for 12 weeks | Inhaled | Tremor, headache, nervousness, cough, nausea |
| Cabrol 2001 ^52^ | 2001 | France | 122 | 27.5 | Preterm labour | Salbutamol (2.5-45 μg/min) for up to 48 h | Intravenous | Tremor, palpitations or tachycardia, headache, anxiety, dyspnea, nausea, vomiting |
| Kissel 2001 ^53^ | 2001 | USA | 30  30 | 35.9  35.5 | Facioscapul-  ohumeral dystrophy | Arm 1: 8.0 mg salbutamol twice daily; Arm 2: 16.0 mg salbutamol twice daily for 52 weeks | Oral | Tremor, palpitations or tachycardia, headache, nervousness, nausea |
| Molimard 2001 ^54^ | 2001 | France | 129 | 39.5 | Asthma | Salbutamol as rescue medication 100 μg/puff for 3 months. | Inhaled | Total AEs, treatment discontinuation |
| Rasul 2001 ^55^ | 2001 | Bangladesh | 45 | 7.3 months | Wheezy | Salbutamol (0.5mg/kg/day) for 48 hours. | Oral | Total AEs, tremor, palpitations or tachycardia, nervousness, vomiting |
| Cydulka 2002 ^56^ | 2002 | USA | 46  48 | 33.0  34.5 | Asthma | Arm 1: salbutamol,7.5 mg per time; arm 2: salbutamol, 2.5 mg, three times | Inhaled | Total AEs, tremor, palpitations or tachycardia, headache, nausea, vomiting |
| Roberts 2003 ^57^ | 2003 | UK | 18 | 3.85 | Asthma | Salbutamol (15 μg/kg over 20 minutes). | Intravenous | Total AEs |
| Busse 2004 ^58^ | 2004 | USA | 79 | 38.4 (13~71) | Asthma | Salbutamol 180 μg four times daily via pMDI for 12 weeks. | Inhaled | Total AEs, headache, cough |
| Mull 2004 ^59^ | 2004 | USA | 32 | 4.1 months | Bronchiolitis | 0.15 mg/kg of 0.5% salbutamol three times for 1 hour | Inhaled | Total AEs |
| Kruse 2005 ^60^ | 2005 | Germany | 16 | 32 (21~49) | Asthma | Salbutamol 600 μg TID via pMDI for 3 days | Inhaled | Tremor, palpitations or tachycardia, headache, nervousness, nausea |
| Langley 2005 ^61^ | 2005 | USA | 31 | 3.32 month | Bronchiolitis | Salbutamol: 1.5 mg for >10 kg, 1.25 mg for > 6 kg and < 10 kg, and 0.75 mg for < 6 kg | Inhaled | Vomiting |
| Aggarwal 2006 ^62^ | 2006 | India | 50 | 41 | Asthma | Salbutamol 0.5mg at intervals of 20 minutes for 1 hour. | Inhaled | Tremor, palpitations or tachycardia |
| Balanag 2006 ^63^ | 2006 | Sweden | 48 | 42 (13~76) | Asthma | Salbutamol 100 μg*2 doses for 3 hours (maximum 1800 μg) | Inhaled | Total AEs, palpitations or tachycardia |
| Donohue 2006 ^64^ | 2006 | USA | 52 | 65.7 | COPD | Racemic albuterol 2.5 mg three times per day for 6 weeks. | Inhaled | Total AEs, treatment discontinuation, tremor, headache, nervousness, cough, dyspnea |
| Kemp 2007 ^65^ | 2007 | USA | 115  117 | 9.3 (6~12)  9.4 (6~12) | Asthma | Arm 1: 1.25 mg salbutamol, arm 2: 0.62 mg salbutamol, three times daily for 4 weeks | Inhaled | Total AEs, palpitations or tachycardia, nausea |
| Donohue 2008 ^66^ | 2008 | USA | 238 | 55.7 | Asthma or COPD | Racemic albuterol 2.5 mg q1–4h for 14 days | Inhaled | Total AEs, severe AEs, treatment discontinuation, palpitations or tachycardia, headache, anxiety |
| Gupta 2008 ^67^ | 2008 | India | 70 | 7.0 months | Bronchiolitis | Salbutamol (0.1 mg/kg/dose) three times daily for 7 days. | Oral | Tremor, vomiting |
| Bigham 2010 ^68^ | 2010 | USA | 22  20 | 7.3  8.2 | Asthma | Arm 1: Heliox-driven salbutamol; arm 2: air driven salbutamol, 15mg/h, for 4 hours. | Inhaled | Palpitations or tachycardia, headache, nervousness, nausea, vomiting |
| Motazedian 2010 ^69^ | 2010 | Iran | 100 | 23.2 | Preterm labour | Salbutamol 0.1 mg (bolus dose) followed by same boluses every 5 minutes | Intravenous | Total AEs, palpitations or tachycardia, anxiety, dyspnea, nausea |
| Direkwatanachai 2011 ^70^ | 2011 | Thailand | 46  48  48 | 9.36  9.25  9.02 | Asthma | Arm 1: salbutamol pMDI, 100μg, 2 puffs for 3 times；Arm 2: salbutamol DPI, 100μg, 1 puff for 6 times; Arm 3: 0.15 mg/kg of salbutamol via oxygen, maximum to 5mg for 3 days | Inhaled | Tremor, palpitations or tachycardia |
| Mangunnegoro  2011 ^71^ | 2011 | Indonesia | 69 | 40.7 | Asthma | One ampoule of salbutamol 3 times every 20 minutes for 1 hour | Inhaled | Palpitations or tachycardia |
| Mattay 2011 ^72^ | 2011 | USA | 152 | 52 | Acute lung injury | Aerosolized salbutamol sulfate (5.0 mg dissolved in saline) every 4 hours for 10 days | Inhaled | Total AEs, treatment discontinuation |
| Smith 2012 ^73^ | 2012 | UK | 162 | 55.8 | Acute respiratory distress syndrome | Salbutamol (15 μg/kg ideal bodyweight per/h) for 7 days | Intravenous | Severe AEs, palpitations or tachycardia |
| Singh 2012 ^74^ | 2012 | Italy | 56 | 40.3 | Asthma | Salbutamol 200 μg pMDI for 1 day | Inhaled | Tremor, dyspnea |
| Lin 2013 ^75^ | 2013 | China | 46 | 3.93 | Asthma | Salbutamol 4 mg bid for 2 weeks | Oral | Tremor |
| Luo 2014 ^76^ | 2014 | China | 112  112 | 42.3  44.2 | Asthma | Arm 1: salbutamol 200 μg single dose domestic pMDI; arm 2: salbutamol 200 μg single dose imported pMDI | Inhaled | Total AEs, palpitations or tachycardia, nausea |
| Raphael 2016 ^77^ | 2016 | USA | 321 | 37.5 | Asthma | Salbutamol DPI 180 μg 4 times/day for 52 weeks | Inhaled | Total AEs, severe AEs, treatment discontinuation, headache, cough |
| LaForce 2017 ^78^ | 2017 | USA | 93 | 8.3 (4~11) | Asthma | Salbutamol DPI 180 μg 4 times daily for 3 weeks | Inhaled | Headache, cough, vomiting |
| Beasley 2019 ^79^ | 2019 | New Zealand, UK, Italy, Australia | 185 | 35.8 | Asthma | Salbutamol (100 μg, two inhalations pMDI) as needed use for 52 weeks | Inhaled | Total AEs, headache, anxiety, cough |
| Katsunuma 2019 ^80^ | 2019 | Japan | 41 | 4.4 | Asthma | Salbutamol (5 mg/kg/h) for 12 hours | Inhaled | Tremor, palpitations or tachycardia |
| Schuh 2020 ^81^ | 2020 | Canada | 407 | 5.0 | Asthma | Salbutamol (5 mg [1 ml]) | Inhaled | Total AEs, severe AEs, headache |

*COPD* chronic obstructive pulmonary disease; *pMDI* pressurized metered dose inhaler; *No.* : number; *DPI* dry powder inhaler; *AE* adverse event
