# Supplementary material for: Safety outcomes of salbutamol: A systematic review and meta‐analysis
Source: Clin Respir J. 2023 Oct 16;17(12):1254–64. doi: 10.1111/crj.13711 (PMC10730473; doi:10.1111/crj.13711)
Supplement: Supplementary file 27 — Table S2. Risk of bias of the included trials. [file CRJ-17-1254-s023.docx]

Supplemental Table 2. Risk of bias of the included trials

| Study | Selection | | | | Comparability | Outcome | | | NOS |
| --- | --- | --- | --- | --- | --- | --- | --- | --- | --- |
|  | Representativeness of the exposed cohort | Selection of the non exposed cohort | Ascertainment  of intervention | Demonstration that outcomes was not present at start of study | Comparability on the basis of the design or analysis | Assessment of outcome | Adequate follow-up duration | Adequate follow-up rate | Overall score |
| Sims 1978 ^24^ | 0 | 0 | 1 | 1 | 0 | 1 | 1 | 1 | 5 |
| Prime 1979 ^25^ | 0 | 0 | 1 | 1 | 0 | 1 | 1 | 1 | 5 |
| Bronsky 1987 ^26^ | 1 | 0 | 1 | 1 | 0 | 1 | 1 | 1 | 6 |
| Assoufi 1989 ^27^ | 0 | 0 | 1 | 1 | 0 | 1 | 1 | 1 | 5 |
| Colacone 1990 ^28^ | 0 | 0 | 1 | 1 | 0 | 1 | 1 | 1 | 5 |
| Pierson 1990 ^29^ | 1 | 0 | 1 | 1 | 0 | 1 | 1 | 1 | 6 |
| Emerman 1991 ^30^ | 0 | 0 | 1 | 1 | 0 | 1 | 1 | 1 | 5 |
| Liippo 1991 ^31^ | 0 | 0 | 1 | 1 | 0 | 1 | 1 | 1 | 5 |
| Pearlman 1992 ^32^ | 1 | 0 | 1 | 1 | 0 | 1 | 1 | 1 | 6 |
| Castle 1993 ^33^ | 1 | 0 | 1 | 1 | 0 | 1 | 1 | 1 | 6 |
| D'Alonzo 1994 ^34^ | 1 | 0 | 1 | 1 | 0 | 1 | 1 | 1 | 6 |
| Steffensen 1995 ^35^ | 1 | 0 | 1 | 1 | 0 | 1 | 1 | 1 | 6 |
| Karpel 1996 ^36^ | 1 | 0 | 1 | 1 | 0 | 1 | 1 | 1 | 6 |
| Khine 1996 ^37^ | 0 | 0 | 1 | 1 | 0 | 1 | 1 | 1 | 5 |
| Shrestha 1996 ^38^ | 0 | 0 | 1 | 1 | 0 | 1 | 1 | 1 | 5 |
| Skoner 1996 ^39^ | 0 | 0 | 1 | 1 | 0 | 1 | 1 | 1 | 5 |
| Auerbach 1997 ^40^ | 1 | 0 | 1 | 1 | 0 | 1 | 1 | 1 | 6 |
| Boulet 1997 ^41^ | 1 | 0 | 1 | 1 | 0 | 1 | 1 | 1 | 6 |
| Jannet 1997 ^42^ | 0 | 0 | 1 | 1 | 0 | 1 | 1 | 1 | 5 |
| Bisits 1998 ^43^ | 0 | 0 | 1 | 1 | 0 | 1 | 1 | 1 | 5 |
| Dobson 1998 ^44^ | 0 | 0 | 1 | 1 | 0 | 1 | 1 | 1 | 5 |
| Kemp 1998 ^45^ | 1 | 0 | 1 | 1 | 0 | 1 | 1 | 1 | 6 |
| Rodrigo 1998 ^46^ | 0 | 0 | 1 | 1 | 0 | 1 | 1 | 1 | 5 |
| Wenzel 1998 ^47^ | 1 | 0 | 1 | 1 | 0 | 1 | 1 | 1 | 6 |
| Bradding 1999 ^48^ | 0 | 0 | 1 | 1 | 0 | 1 | 1 | 1 | 5 |
| Campbell 1999 ^49^ | 1 | 0 | 1 | 1 | 0 | 1 | 1 | 1 | 6 |
| Nelson 1999 ^50^ | 1 | 0 | 1 | 1 | 0 | 1 | 1 | 1 | 6 |
| Bensch 2001 ^51^ | 1 | 0 | 1 | 1 | 0 | 1 | 1 | 1 | 6 |
| Cabrol 2001 ^52^ | 1 | 0 | 1 | 1 | 0 | 1 | 1 | 1 | 6 |
| Kissel 2001 ^53^ | 0 | 0 | 1 | 1 | 0 | 1 | 1 | 1 | 5 |
| Molimard 2001 ^54^ | 1 | 0 | 1 | 1 | 0 | 1 | 1 | 1 | 6 |
| Rasul 2001 ^55^ | 0 | 0 | 1 | 1 | 0 | 1 | 1 | 1 | 5 |
| Cydulka 2002 ^56^ | 0 | 0 | 1 | 1 | 0 | 1 | 1 | 1 | 5 |
| Roberts 2003 ^57^ | 0 | 0 | 1 | 1 | 0 | 1 | 1 | 1 | 5 |
| Busse 2004 ^58^ | 1 | 0 | 1 | 1 | 0 | 1 | 1 | 1 | 6 |
| Mull 2004 ^59^ | 0 | 0 | 1 | 1 | 0 | 1 | 1 | 1 | 5 |
| Kruse 2005 ^60^ | 0 | 0 | 1 | 1 | 0 | 1 | 1 | 1 | 5 |
| Langley 2005 ^61^ | 0 | 0 | 1 | 1 | 0 | 1 | 1 | 1 | 5 |
| Aggarwal 2006 ^62^ | 0 | 0 | 1 | 1 | 0 | 1 | 1 | 1 | 5 |
| Balanag 2006 ^63^ | 0 | 0 | 1 | 1 | 0 | 1 | 1 | 1 | 5 |
| Donohue 2006 ^64^ | 0 | 0 | 1 | 1 | 0 | 1 | 1 | 1 | 5 |
| Kemp 2007 ^65^ | 1 | 0 | 1 | 1 | 0 | 1 | 1 | 1 | 6 |
| Donohue 2008 ^66^ | 1 | 0 | 1 | 1 | 0 | 1 | 1 | 1 | 6 |
| Gupta 2008 ^67^ | 1 | 0 | 1 | 1 | 0 | 1 | 1 | 1 | 6 |
| Bigham 2010 ^68^ | 0 | 0 | 1 | 1 | 0 | 1 | 1 | 1 | 5 |
| Motazedian 2010 ^69^ | 1 | 0 | 1 | 1 | 0 | 1 | 1 | 1 | 6 |
| Direkwatanachai 2011 ^70^ | 1 | 0 | 1 | 1 | 0 | 1 | 1 | 1 | 6 |
| Mangunnegoro 2011 ^71^ | 1 | 0 | 1 | 1 | 0 | 1 | 1 | 1 | 6 |
| Mattay 2011 ^72^ | 1 | 0 | 1 | 1 | 0 | 1 | 1 | 1 | 6 |
| Smith 2012 ^73^ | 1 | 0 | 1 | 1 | 0 | 1 | 1 | 1 | 6 |
| Singh 2012 ^74^ | 1 | 0 | 1 | 1 | 0 | 1 | 1 | 1 | 6 |
| Lin 2013 ^75^ | 0 | 0 | 1 | 1 | 0 | 1 | 1 | 1 | 5 |
| Luo 2014 ^76^ | 1 | 0 | 1 | 1 | 0 | 1 | 1 | 1 | 6 |
| Raphael 2016 ^77^ | 1 | 0 | 1 | 1 | 0 | 1 | 1 | 1 | 6 |
| LaForce 2017 ^78^ | 1 | 0 | 1 | 1 | 0 | 1 | 1 | 1 | 6 |
| Beasley 2019 ^79^ | 1 | 0 | 1 | 1 | 0 | 1 | 1 | 1 | 6 |
| Katsunuma 2019 ^80^ | 0 | 0 | 1 | 1 | 0 | 1 | 1 | 1 | 5 |
| Schuh 2020 ^81^ | 1 | 0 | 1 | 1 | 0 | 1 | 1 | 1 | 6 |
